# Colloidal Photonic Crystals of Reusable Hydrogel Microparticles for Sensor and Laser Applications

**DOI:** 10.1021/acsami.1c16500

**Published:** 2021-11-25

**Authors:** Naoto Iwata, Takeru Koike, Kaya Tokuhiro, Ryu Sato, Seiichi Furumi

**Affiliations:** Department of Applied Chemistry, Faculty of Science, Tokyo University of Science, 1-3 Kagurazaka, Shinjuku, Tokyo 162-8601, Japan

**Keywords:** photonic crystal, hydrogel, microparticle, reflection, temperature response, sensor, laser, photonic band gap, reusability

## Abstract

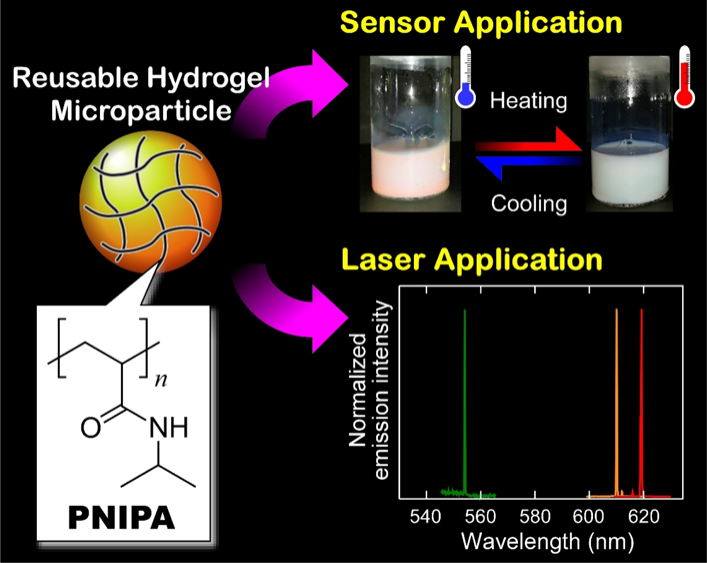

Although a wide variety of techniques have been developed to date for the fabrication of high-quality colloidal photonic crystals (CPCs) using monodisperse silica and polystyrene microparticles, poly(*N*-isopropylacrylamide) (PNIPA) hydrogel microparticles have rarely been utilized for the preparation of active CPCs despite the intriguing feature of temperature-responsive volume changes. This report describes the promising potential abilities of PNIPA hydrogel microparticles for sensor and laser applications. Monodisperse PNIPA hydrogel microparticles were synthesized by emulsion polymerization, and the microparticle diameter was finely controlled by adjusting the surfactant concentration. Such hydrogel microparticles spontaneously formed uniform CPCs with visible Bragg reflection even in fluid suspensions. The addition of small amounts of ionic substances into the centrifuged and deionized CPC suspensions enabled the on-demand color switching between Bragg reflection and white turbidity with temperature, leading to temperature- and ion-sensing applications. Moreover, our expanding experiments successfully demonstrated the optically excited laser action with a single and narrow peak from CPC suspensions with light-emitting dyes by the photonic band gap effect. After the light-emitting dyes were simply removed from the CPC suspensions by centrifugation, the purified PNIPA hydrogel microparticles were permanently reusable as the CPC laser microcavities to generate the laser action at other wavelengths using different dyes. This study contributes the circular economy concept using reusable hydrogel microparticles for the realization of a sustainable society.

## 1 Introduction

Photonic crystals (PCs) have attracted considerable interest as a promising paradigm in the photonics research studies from both fundamental and technological viewpoints because of their outstanding features to confine and manipulate photons in a spatiotemporal way.^1,2^ The PCs are the periodically modulated microarchitectures of different dielectric materials with the intervals comparable to the lengths of electromagnetic waves. The hierarchical structures cause the appearance of forbidden regions for photons in the dispersion spectrum. At present, these spectral regions are known as photonic band gaps (PBGs). The PC systems have intriguing abilities for the on-demand control of photons as well as the enhancement of photon–matter interactions in spatial confinement, arising from their extremely low group velocities near or within the PBG regions. Consequently, novel PCs enable the further technological developments for next-generation all-optical circuits with the PBGs such as microcavities, waveguides, and so forth. Based on their periodic dimensionality, the PCs can be categorized into three types: 1D-, 2D-, and 3D-PCs. Although some 1D- and 2D-PCs are commercially available through limited fabrication procedures, mass production of 3D-PCs should be resolved for the practical applications. Moreover, 3D-PCs have an outstanding optical property to fully prevent the electromagnetic wave propagation within a specific PBG region and in any direction to PCs by the complete PBG.^3−6^ To date, significant efforts have been made to establish the diverse methodologies to fabricate 3D-PCs with PBGs in the infrared wavelength regime using top-down stereolithographic techniques involving laser or electron beams. For optoelectronic applications, the periodicity in 3D-PCs should be designed and constructed on several hundred nanometers that are compatible with the optical wavelength regime. However, these top–down techniques have serious bottlenecks such as the limited processing resolution and high-cost equipment in order to downsize the periodicity of 3D-PCs to several hundred nanometers. Consequently, significant progress has been made on a wide variety of procedures to prepare large-sized 3D-PCs with visible-range PBGs by bottom–up techniques.

Over the past few decades, the self-assembly of colloidal microparticles has proven to be a rational bottom–up technique for fabricating 3D-PCs with the PBGs in the optical wavelength regime.^7−19^ By utilizing interparticle electrostatic interaction, the monodisperse colloidal microparticles as building blocks of 3D-PCs exhibit spontaneous accumulation of 3D ordered hierarchical structures, that is, colloidal PCs (CPCs) or colloidal crystals, from the fluid suspensions. Therefore, the CPCs are readily prepared as 3D-PCs without any complicated fabrication process. Notably, when the colloidal microparticles with diameters of several hundred nanometers are adopted for the CPC fabrication, the PBGs can be observed as Bragg reflection colors by the naked eye. The reflection wavelength (λ_ref_) is approximately determined by the following geometric equation with the combination of Bragg and Snell laws^20^
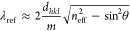
1where *d*_*hkl*_ represents the lattice space in the [*hkl*] direction, *m* is the diffraction order, *n*_eff_ is the effective refractive index of materials, and θ is the incident angle from the horizontal plane, that is, the Bragg angle. In general, the reflection characteristics of CPC films arise from the light reflection by the (111) plane of the face-centered cubic lattice of colloidal microparticles. Considering the filling ratio (*f*) of the colloidal microparticles in the CPCs, *n*_eff_ is determined as follows^20^
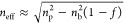
2where *n*_p_ and *n*_b_ denote the refractive indices of materials in microparticles and the background encompassing them, respectively. According to eqs 1 and 2, the reflection wavelength can be precisely tuned by controlling the refractive indices, diameter, and filling ratio of monodisperse microparticles.

Previously, numerous studies have shown various techniques of fabricating high-quality CPCs using monodisperse microparticles of silica and polystyrene (PS), which are relatively hard and inert to external stimuli. These colloidal microparticles are commercially available because they are mass-produced by a number of companies.^7^ To fabricate active CPCs for photonic applications such as colorimetric sensors that are responsive to environmental stimuli, the general fabrication strategy is to fill the void space between the 3D ordered colloidal microparticles of CPCs with soft materials such as hydrogels, elastomers, liquid crystals, and so forth. In this context, the active CPC films of microparticles stabilized with stimuli-responsive hydrogels of poly(*N*-isopropylacrylamide) (PNIPA) and its derivatives are one of the most frequently investigated CPC systems.^21−27^ This is because the PNIPA hydrogels swell and shrink around the lower critical solution temperature of ∼33 °C in water through the reversible volume changes and thus absorb and expel large amounts of water in response to changes in temperature, respectively.^28−30^ Such active CPC systems with soft polymer hydrogels allow us to quantitatively monitor external stimuli through the visual alterations in their reflection colors, originating from the fluctuations in the interparticle distance, that is, *d*_*hkl*_ noted in eq 1.

Thus, the monodisperse silica and PS microparticles are widely adopted to fabricate the CPCs with visible reflection properties. In contrast, although polymer hydrogels show the interesting feature of reversible volume changes induced by temperature, monodisperse microparticles of PNIPA hydrogels have scarcely ever been utilized for the preparation of active CPCs as compared to monodisperse silica and PS microparticles. Pelton and Chibante synthesized for the first time aqueous suspensions of cross-linked PNIPA hydrogel microparticles with diameters of ∼500 nm by surfactant-free emulsion polymerization.^31^ These hydrogel microparticles have an important advantage over bulk hydrogels: the swelling speeds of spherical hydrogel particles are proportional to the square of the sizes of these particles.^32^ Thus, hydrogel microparticles can respond to external stimuli considerably faster than bulk hydrogels.^33^ Most of the previous studies on polymer hydrogel microparticles have been focused on the biological applications in immunoassay, tissue engineering, and drug delivery systems. However, only a limited number of studies have been reported on the use of temperature-responsive monodisperse microparticles of PNIPA hydrogels with diameters of ∼200 nm for the fabrication of active CPCs with reflection propensities in the visible wavelength range.^21,34−36^ Therefore, the fabrication of CPCs using PNIPA hydrogel microparticles and their practical applications still remain challenging topics.

In this study, we developed novel capabilities of polymer hydrogel microparticles leading to not only sensor but also laser applications. Monodisperse microparticles of the PNIPA hydrogel have an intrinsic feature to self-assemble into uniform and large-sized CPCs with visible reflection even in aqueous suspension media. With regard to the temperature-responsive phenomena of reflection properties from CPC suspensions of PNIPA hydrogel microparticles, it was found that the concentration of ionic components in CPC suspensions is of prime importance. After the PNIPA hydrogel microparticles were purified by centrifugation and deionization, the corresponding CPC suspensions exhibited no alterations in the reflection colors by temperature variation; however, the microparticles significantly shrunk due to the temperature-induced volume change of the PNIPA hydrogel, as confirmed by dynamic light scattering (DLS) measurements. In contrast, the addition of small amounts of ionic substances into the centrifuged and deionized CPC suspensions of PNIPA hydrogel microparticles enabled the temperature-induced reversible switching between reflection-colored and white turbid states, leading to temperature- and ion-sensing applications. Interestingly, the CPC suspensions of PNIPA hydrogel microparticles exhibited a narrow Bragg reflection peak with the relatively high reflectance of ∼60%. To the best of our knowledge, although many studies have hitherto shown the laser action by utilizing the PBG of CPC films doped with light-emitting materials,^37−46^ there has been no demonstration of laser action using the CPC suspensions of PNIPA hydrogel microparticles. Taking advantage of the prominent reflection characteristic, we successfully demonstrated the optically excited laser action from aqueous CPC suspensions of PNIPA hydrogel microparticles containing light-emitting dyes. A single and narrow laser peak appeared near the longer-wavelength edge of the reflection band by optical excitation, arising from the PBG effect of the CPC suspension. The laser peak could be reversibly switched by adjusting the temperature through the self-assembly/disassembly of CPC structures. Furthermore, the light-emitting dyes were readily removed from the CPC suspensions by centrifugation so that the PNIPA microparticles were purifiable and reusable as CPC laser microcavities to generate the laser action at other wavelengths; this was achieved not only by changing the different light-emitting dyes in the suspensions but also by tuning the reflection band on the basis of microparticle concentration. From the ecological perspective, these purifiable and reusable materials would be greatly advantageous in overcoming the serious issues, such as mass production, mass consumption, and waste mismanagement, related to the use of finite resources worldwide in the development of a sustainable society.

## 2 Experimental Section

### 2.1 Synthesis of Monodisperse PNIPA Hydrogel Microparticles

All the reagents (Fujifilm Wako Pure Chemical Co., Ltd.) were used as received. Ultrapure water was obtained using a water purification system (Direct-Q UV 5, Merck Millipore). Monodisperse PNIPA hydrogel microparticles were synthesized by the emulsion polymerization of *N*-isopropylacrylamide (NIPA) as a primary monomer with *N*,*N*′-methylenebis(acrylamide) (BIS) as a cross-linker in the presence of sodium dodecyl sulfate (SDS) as a surfactant. In this study, the SDS concentrations were varied in the range between 0.17 and 2.31 mM (Table 1). A typical procedure of emulsion polymerization at the SDS concentration at 1.16 mM is described as follows (Table 1, sample code: **PNIPA-5**).

**Table 1:** Amounts and Concentrations of SDS during the Emulsion Polymerization of NIPA and BIS and Size Properties of the Resultant PNIPA Hydrogel Microparticles^a^

sample code	amount of SDS (g)	SDS concentration (mM)	mean diameter at 25 °C (nm)^b^	CV in diameter at 25 °C (%)^b^
**PNIPA-1**	0.03	0.17	383	10.3
**PNIPA-2**	0.05	0.29	356	11.3
**PNIPA-3**	0.10	0.58	307	10.8
**PNIPA-4**	0.15	0.87	269	10.1
**PNIPA-5**	0.20	1.16	248	10.2
**PNIPA-6**	0.25	1.44	217	10.7
**PNIPA-7**	0.30	1.73	206	11.3
**PNIPA-8**	0.40	2.31	173	11.4

a Polymerization was performed using 30 g of NIPA (0.27 mol) and 2.0 g of BIS (13 mmol) as the PNIPA hydrogel precursors in 600 mL of ultrapure water using 0.03–0.40 g of SDS (0.10–1.39 mmol), as mentioned in Section 2.1.

b These values were calculated by DLS measurements at 25 °C.

At first, 30 g of NIPA (0.27 mol), 2.0 g of BIS (13 mmol), and 0.20 g of SDS (0.69 mmol) were added to 600 mL of ultrapure water. After this reaction solution was stirred at 250 rpm using a mechanical stirrer at room temperature under a flow of nitrogen gas for 1 h to purge oxygen, the temperature was elevated to 70 °C using a mantle heater for 1 h. To initiate the polymerization, a solution of 0.30 g of potassium peroxodisulfate (KPS) (1.1 mmol) in 15 mL of ultrapure water was prepared in advance. After the aqueous solution of KPS was portion-wise added into the reaction solution including NIPA, BIS, and SDS, the stirring at 70 °C was prolonged for additional 4 h. Subsequently, the polymerization was terminated by exposing the reaction solution to air, followed by gradual cooling to room temperature. The aqueous suspension of PNIPA hydrogel microparticles was filtered through a cellulose filter of no. 5A and two kinds of polytetrafluoroethylene membrane filters with the pore sizes of ∼5.0 and ∼0.2 μm to remove the aggregated microparticles.

Herein, the resultant PNIPA hydrogel microparticles were purified by dialysis or a combination of centrifugation and deionization. The detailed procedures are explained in the following section.

### 2.2 Purification of PNIPA Hydrogel Microparticles by Dialysis

The as-prepared suspension of PNIPA hydrogel microparticles, as described in Section 2.1, was purified by dialysis in ultrapure water for 10 days using visking tubes with pore sizes of ∼5 nm and a molecular weight cutoff of 1.2–1.4 × 10^4^. During the dialysis, the water outside the visking tubes was replaced with fresh ultrapure water every 12 h to remove the contaminants from the suspension.

### 2.3 Purification of PNIPA Hydrogel Microparticles by the Combination of Centrifugation and Deionization

As another purification, the aqueous suspension of PNIPA hydrogel microparticles, as stated in Section 2.1, was separated using a tabletop ultra-centrifuge (Optima MAX-XP, Beckman Coulter) operated at the relative centrifugal force of 233,000*g* for 30 min. After the aqueous supernatant was removed from the centrifuge tube, the sediments comprising PNIPA hydrogel microparticles were redispersed in fresh ultrapure water. At this time, the removed volume of the supernatant was adjusted to be identical to the added volume of ultrapure water for the redispersion of the PNIPA hydrogel microparticles. Thus, the PNIPA hydrogel microparticles were centrifuged and redispersed in ultrapure water three times to remove the unreacted PNIPA precursors and byproducts. Finally, negligible amounts of ionic contaminants in the aqueous suspension of PNIPA hydrogel microparticles were eliminated by deionization using ion-exchange resin beads (Bio-Rex MSZ 501D, Bio-Rad) for 24 h.

The physical properties of PNIPA hydrogel microparticles, purified by dialysis or the combination of centrifugation and deionization, and their CPC suspensions were characterized according to the procedures mentioned in the Supporting Information.

### 2.4 Preparation of CPC Suspensions of PNIPA Hydrogel Microparticles for the Laser Action

At first, PNIPA hydrogel microparticles of **PNIPA-5** were purified by the combination of centrifugation and deionization. To generate the laser action, the aqueous suspension of **PNIPA-5** was doped with three types of water-soluble light-emitting dyes (Exciton, Inc.): Rhodamine B (**RhB**), Pyrromethene 556 (**Py556**), and Sulforhodamine B (**SrB**). All the dyes were readily dissolved in water because of their hydrophilic and ionic moieties. By considering our previous experimental results, the reflection band of the CPC structure should overlap with the broad spontaneous emission (fluorescence) band of the light-emitting dye in order to efficiently generate the laser action.^20,37−49^ Therefore, the reflection peak wavelength was tuned by controlling the appropriate concentration of PNIPA hydrogel microparticles in suspension. Thereafter, **RhB**, **Py556**, or **SrB** was dissolved into the aqueous CPC suspension of PNIPA hydrogel microparticles. Finally, the CPC suspension of PNIPA microparticles with the light-emitting dye was placed between a pair of glass substrates with the gap of ∼100 μm.

To explore the laser action at the other wavelengths, the CPC suspension of PNIPA hydrogel microparticles with the light-emitting dye was separated by ultra-centrifugation. The suspension was divided into two layers: an aqueous supernatant containing the light-emitting dye and a sediment of PNIPA hydrogel microparticles. After carefully removing the supernatant, the PNIPA hydrogel microparticles were redispersed in fresh ultrapure water. The light-emitting dye was approximately eliminated by the combination of centrifugation and redispersion in a repeated manner. To analyze the remaining light-emitting dye in the supernatant after the centrifugation, absorption spectra were acquired using a photodiode array spectrophotometer (Agilent 8453, Agilent Technologies). Finally, the aqueous suspension of PNIPA hydrogel microparticles was prepared at a certain concentration, followed by the addition of a different light-emitting dye for the generation of the laser action at another wavelength.

### 2.5 Emission Spectral Measurements for the Laser Action

Emission properties of the CPC suspensions of PNIPA hydrogel microparticles with light-emitting dyes were evaluated by optical excitation with an incident light at 500 or 532 nm, which was tuned by an optical parametric oscillator (OPO) (Surelite OPO Plus, Continuum) combined with a *Q*-switched Nd:yttrium aluminum garnet (Nd:YAG) laser beam (Surelite I-10, Continuum). An original setup of laser experiments was constructed to measure the local emission spectra of the CPC suspension by the exposure of the excitation light through a motorized illuminator of a microscope.^43,50^ The pulse duration of the incident excitation light was ∼6 ns, and the repetition frequency was tuned to 10 Hz. The optical excitation energy, which was precisely controlled by the combination of a half-wave plate, a Glan-Laser polarizing prism, and neutral-density filters, was monitored using an energy analyzer equipped with a pyroelectric sensor (PE-9, Ophir). The excitation light propagated along the surface normal of CPC suspensions sandwiched between two glass plates and was focused on the CPC suspensions through a microscopic objective lens with a long working distance of 25 mm (SLMPLN 20×, Olympus) to obtain a circular spot with the diameter of ∼40 μm. Emission spectra in the collinearly transmitted direction were recorded using a spectrometer (SR-303i, Andor Technology) paired with a front-illuminated charge-coupled device detector (iDus DU420A, Andor Technology).

## 3 Results and Discussion

### 3.1 Synthesis of PNIPA Hydrogel Microparticles

Preliminary experiments were performed to investigate the role of BIS in the synthesis of monodisperse PNIPA hydrogel microparticles. When the emulsion polymerization trials of NIPA were conducted in the absence of BIS, the resultant PNIPA hydrogel microparticles showed high values in the coefficient of variation (CV) in the diameter, corresponding to the ratio of the standard deviation to the average diameter. For example, when the PNIPA hydrogel microparticles were synthesized by the emulsion polymerization of 30 g of NIPA (0.27 mol) in 600 mL of ultrapure water at the SDS concentration of 1.16 mM, the diameter and its CV were estimated to be ∼260 nm and 43%, respectively. These results suggested that these PNIPA hydrogel microparticles may not be suitable for the fabrication of CPC structures by the self-assembly owing to the substantially high CV in the diameter, that is, the broad distribution of the particle diameter.

In contrast, when BIS was employed in the emulsion polymerization of NIPA, monodisperse PNIPA hydrogel microparticles with relatively low CVs in the diameter were acquired by the formation of a spherical network of the PNIPA hydrogel with the aid of BIS. Thus, the cross-linking agent of BIS is indispensable to the synthesis of monodisperse PNIPA hydrogel microparticles with diameters of several hundred nanometers. However, the temperature-dependent volume change ratios of PNIPA hydrogels decrease when large amounts of cross-linking agents are introduced into the hydrogels and vice versa.^51,52^ To optimize the trade-off relationship between the CVs in the diameter of PNIPA microparticles and large temperature-dependent volume change ratios, we investigated for the adequate amount of BIS during the emulsion polymerization of NIPA. After several synthesis trials, it turned out that 2.0 g of BIS (13 mmol) is the most suitable for the emulsion polymerization of 30 g of NIPA (0.27 mol) with SDS in 600 mL of ultrapure water to produce monodisperse PNIPA hydrogel microparticles with both relatively low CVs in the diameter and large temperature-dependent volume change ratios (Supporting Information, Figure S1).

Considering the results of the preliminary experiments, the emulsion polymerizations of 30 g of NIPA (0.27 mol) and 2.0 g of BIS (13 mmol) as the PNIPA precursors were carried out in 600 mL of ultrapure water at SDS concentrations ranging from 0.17 to 2.31 mM. As detailed in Table 1, a series of PNIPA hydrogel microparticles were synthesized at various SDS concentrations; these specimens are hereinafter referred using the sample codes as from **PNIPA-1** to **PNIPA-8**. The mean diameters of these PNIPA hydrogel microparticles at 25 °C, determined by DLS measurements, were finely controlled between 173 and 383 nm by regulating the SDS concentration during the emulsion polymerization (Table 1). When the SDS concentration was increased from 0.17 to 2.31 mM, the mean diameter of PNIPA hydrogel microparticles continuously decreased from 383 to 173 nm. Similarly, the previous studies on the synthesis of monodisperse PS microparticles have shown that the diameters become smaller with the increase in the surfactant concentration during the emulsion polymerization of the styrene monomer.^49,52^ Nevertheless, the experimental results obtained herein were also supported by those reported in previous studies on micelles. It was surmised that the SDS molecules self-assemble to form micellar structures in water, wherein the monodisperse PNIPA hydrogel microparticles are produced by the polymerization of NIPA and BIS. Therefore, the mean diameters of PNIPA hydrogel microparticles may be determined based on the diameters of micelles formed by surfactants. According to previous studies reported on the formation of micelles by surfactants, the micelle sizes reduce with the increase in the concentrations of surfactants.^53^ These facts indicated that the SDS molecules at high concentrations form smaller micelles in water, thereby resulting in PNIPA hydrogel microparticles with smaller diameters. Thus, the diameters of PNIPA hydrogel microparticles are governed by the concentration of SDS employed in the emulsion polymerization.

**Figure 1:**
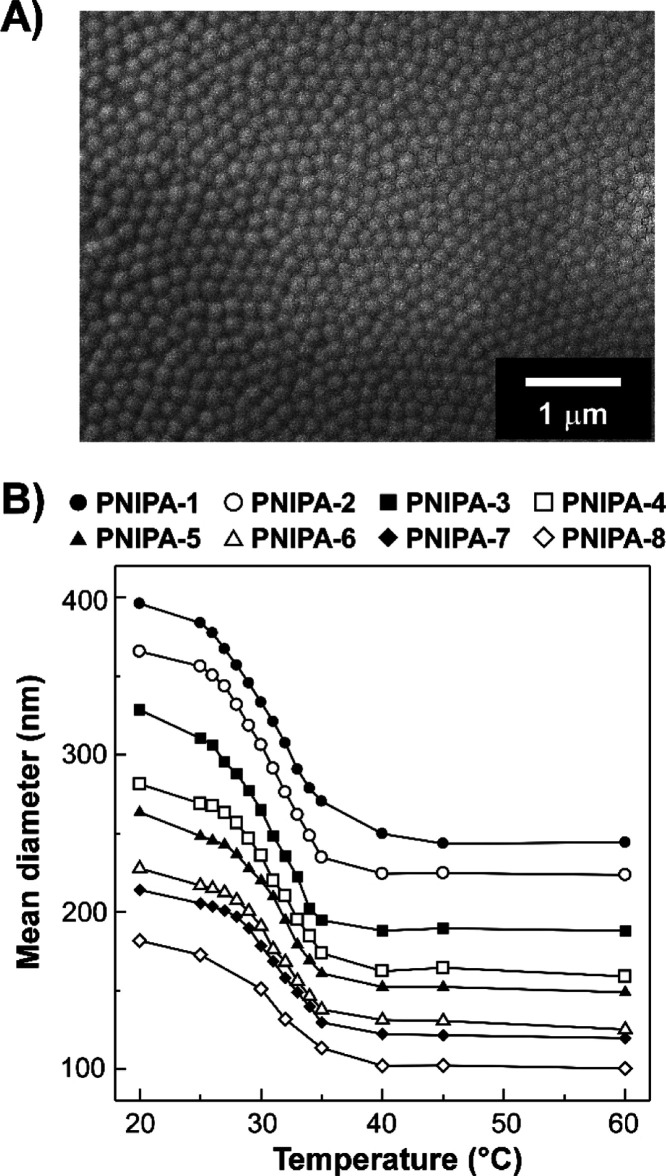
(A) SEM image of **PNIPA-5** collapsed on a substrate after drying. As detailed in Table 1, **PNIPA-5** was synthesized by emulsion polymerization at the SDS concentration of 1.16 mM. The white scale bar represents 1 μm. (B) Changes in the mean diameters of PNIPA hydrogel microparticles, analyzed by DLS measurements, as a function of temperature. These PNIPA hydrogel microparticles, **PNIPA-1**–**PNIPA-8**, were prepared by emulsion polymerization at SDS concentrations of 0.17–2.31 mM, respectively, as listed in Table 1.

**PNIPA-1**–**PNIPA-8** exhibited relatively low CVs in diameter of ∼10% regardless of the SDS concentration (Table 1). The emulsion polymerization of NIPA and BIS with SDS yielded monodisperse PNIPA hydrogel microparticles with the diameters of several hundred nanometers. Although scanning electron microscopy (SEM) can be employed to visualize the actual morphologies, including sizes and shapes, of microparticles at microscopic levels, it is not straightforward to analyze the morphologies of PNIPA hydrogel microparticles due to the swollen state with water. Accordingly, the SEM observation of PNIPA hydrogel microparticles was attempted after drying, as described in the Supporting Information. After an aqueous suspension of **PNIPA-5** was loaded on a silicon wafer as a substrate, water of the suspension was thoroughly evaporated by mild drying. Interestingly, the SEM image verified that the microparticles probably collapse on the substrate surface upon the shrinkage of PNIPA networks after drying, all the dried microparticles have well-defined spherical shapes and almost uniform diameters of ∼170 nm, as shown in Figure 1A. Likewise, the other PNIPA hydrogel microparticles with different diameters were observed by SEM. For instance, although **PNIPA-4**, **PNIPA-6**, and **PNIPA-7** also collapsed on the substrates after the sample preparation process for SEM observation similar to **PNIPA-5**, the spherical shapes were recognizable (Supporting Information, Figure S2). Moreover, the diameters of dried **PNIPA-4**, **PNIPA-6**, and **PNIPA-7** were calculated to be ∼180, ∼150, and ∼140 nm, respectively.

### 3.2 Temperature-Responsive Diameters of PNIPA Hydrogel Microparticles

Hydrodynamic diameters of PNIPA hydrogel microparticles in the aqueous suspensions were analyzed in the temperature range of 20–60 °C by DLS. As presented in Figure 1B, the diameters of **PNIPA-1**–**PNIPA-8** were clearly dependent on the temperature. All the PNIPA hydrogel microparticles showed the gradual decrease in diameter with the increase in the temperature from 20 to 35 °C due to their temperature-responsive volume changes. When the temperature exceeded 40 °C, the diameters had almost constant values, which were ∼0.58-fold smaller than those obtained at 20 °C. For instance, the diameter of **PNIPA-5** decreased from 263 to 149 nm in an inverse sigmoidal manner with the increase in temperature from 20 to 60 °C (Figure 1B, closed triangles). Such changes in the diameters of PNIPA microparticles were similar to those observed for bulk PNIPA hydrogels; however, the critical changing temperatures were slightly shifted owing to the different contents of cross-linking agents in PNIPA hydrogels.^23,28^ It should be stressed that the CVs in diameter of **PNIPA-5** maintain a value of ∼10% over a wide temperature range of 20–60 °C, as revealed by DLS measurements (Supporting Information, Table S1). After successive cooling from 60 to 20 °C, the PNIPA hydrogel microparticles were swelled with water again so as to revert to their initial diameters. Moreover, the diameters of PNIPA hydrogel microparticles reversibly changed with repeated cycles of heating and cooling processes, pronouncing the stability of dispersions of monodisperse PNIPA hydrogel microparticles in water at both the swollen and shrunken states.

### 3.3 CPC Formation of PNIPA Hydrogel Microparticles in Suspensions

**Figure 2:**
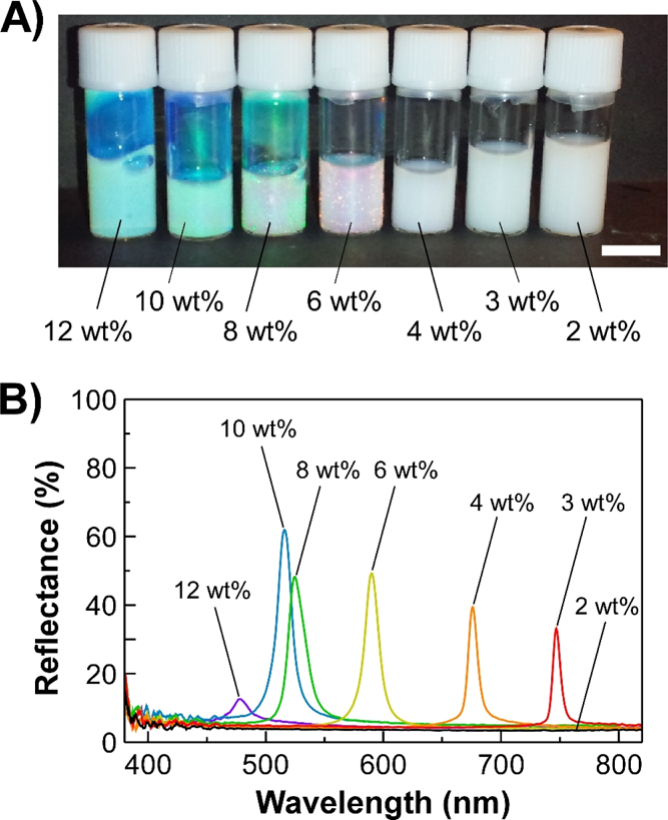
Image (A) and reflection spectra (B) of the aqueous suspensions of **PNIPA-5** whose concentrations were adjusted in the range of 2–12 wt %. Both images and spectra were recorded at room temperature. The white scale bar in the image represents 10 mm.

Interestingly, the PNIPA hydrogel microparticles showed the spontaneous formation of CPC structures with reflection colors even in aqueous suspensions after purification by dialysis. Taking account of the filling ratio of colloidal microparticles, corresponding to *f* in eq 2, the dialyzed suspensions of **PNIPA-5** were prepared at particle concentrations in the range from 2 to 12 wt %, as shown in Figure 2. As is evident from the images of **PNIPA-5** suspensions acquired at room temperature, the visual appearances showed a white color at 2–4 wt %, red at 6 wt %, and green at 8–12 wt % (Figure 2A). Nevertheless, the reflection spectral results were slightly different from the findings deduced from these images (Figure 2B). At the **PNIPA-5** concentration of 2 wt %, no reflection peak was observed for the suspension (Figure 2B, black line), resulting in the visual appearance of white turbidity owing to Mie scattering. It was inferred that the PNIPA hydrogel microparticles hardly assemble into the CPC structures because of the very low **PNIPA-5** concentration diluted with water. In contrast, in the reflection spectra of 3 and 4 wt % **PNIPA-5** suspensions, the characteristic reflection peaks appeared at ∼740 and ∼680 nm, respectively (Figure 2B, red and orange lines). Therefore, the reflection colors could not be observed in the images due to the near-infrared Bragg reflection. With the increase in the **PNIPA-5** concentration from 6 to 10 wt %, the reflection peak shifted to a shorter wavelength from 580 to 510 nm, accompanied by the gradual enhancement of the light reflectance from 50 to 60%, respectively (Figure 2B, yellow, green, and blue lines). These results implied that uniform CPC structures are formed in the 6–10 wt % suspensions of **PNIPA-5**. When the **PNIPA-5** concentration was further increased to 12 wt %, the Bragg reflection abruptly weakened to the reflectance of ∼10% (Figure 2B, violet line) due to the polycrystalline structures of the PNIPA hydrogel microparticles. This was most probably caused by the deterioration in the CPC structure of **PNIPA-5** due to the collision of microparticles with each other due to the high **PNIPA-5** concentration. Although the 12 wt % suspension of **PNIPA-5** showed a low light reflectance of ∼10% by the reflection spectral measurement in the direction of regular reflection (Supporting Information, Experimental Details), a blue color was clearly observed for the photographic image, arising from not only regular but also diffused light reflection components in the image (Figure 2A). Thus, the 6–10 wt % suspensions of PNIPA hydrogel microparticles would be suitable for the preparation of CPC structures with a relatively high reflectance of visible light by the self-assembly of microparticles.

**Figure 3:**
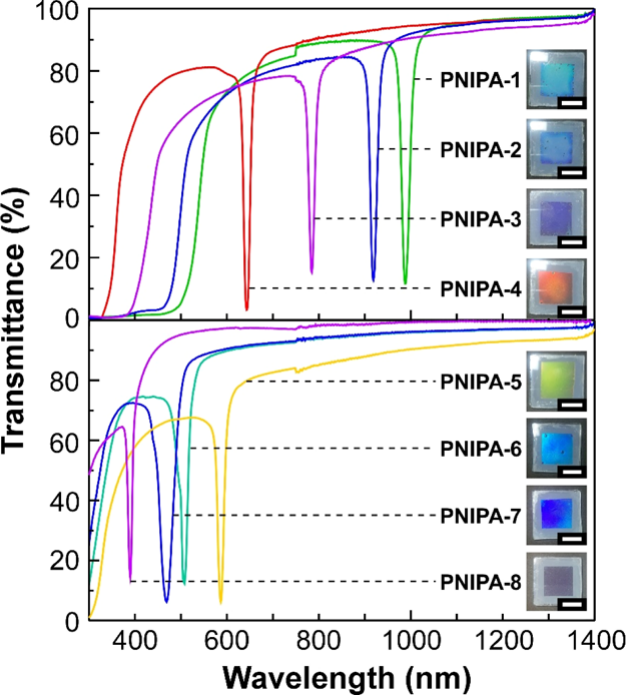
Transmission spectra of the aqueous suspensions of **PNIPA-1**–**PNIPA-4** (upper panel) and **PNIPA-5**–**PNIPA-8** (lower panel) at room temperature. All the microparticle concentrations were adjusted to 6 wt %, and the suspensions were sandwiched between two glass substrates with an internal gap of ∼100 μm. Insets represent the reflection images with white scale bars of 5 mm.

Further investigations demonstrated that the reflection peak can be controlled using **PNIPA-1**–**PNIPA-8** with different diameters, as shown in Figure 3. As mentioned above, all the PNIPA hydrogel microparticles synthesized herein showed the monodispersity of diameter, corresponding to the relatively low CVs in diameter of ∼10% (Table 1). Therefore, the monodisperse PNIPA hydrogel microparticles are considered to spontaneously form the CPC structures in ∼6 wt % suspensions. Reflection peaks in the transmission spectra of the CPC suspensions of **PNIPA-1**–**PNIPA-8** appeared at different wavelengths in a wide spectral range from ∼380 to ∼980 nm, which entirely covered the visible and even the near-infrared wavelength regions. The sharp and narrow peaks of the Bragg reflection most probably originated from not only the well-ordered CPC structures of PNIPA hydrogel microparticles in aqueous suspensions but also the low contrast of refractive indices between the microparticle and water. Upon changing the PNIPA hydrogel microparticles from **PNIPA-1** to **PNIPA-8**, the reflection peak wavelength continuously shifted from 980 to 380 nm according to the decrease in the microparticle diameter. Such blue shifts in the reflection peaks stemmed from the geometric decrease in the interparticle distance, corresponding to *d*_*hkl*_ in eq 1, caused by the decrease in the microparticle diameter. Unexpectedly, although the CPC suspensions of **PNIPA-1**, **PNIPA-2**, and **PNIPA-3** showed reflection peaks at 980, 920, and 780 nm, respectively, the reflection colors were observed as blue or violet (Figure 3, insets). It is presumable that the reflection colors originate from the Rayleigh scattering or second-order Bragg reflection but not the primary Bragg reflection. Indeed, these results were supported by the broad bands below 500 nm in the transmission spectra of **PNIPA-1**–**PNIPA-3**. In contrast, the CPC suspensions of **PNIPA-4**–**PNIPA-8** displayed uniform reflection colors of red, green, and blue according to the Bragg reflection peaks (Figure 3, inset).

To evaluate the statistical uniformity of reflection characteristics on a macroscopic area, transmission spectral imaging measurements using a hyperspectral camera were performed, as described in the Supporting Information. For a 10 wt % CPC suspension of **PNIPA-4**, the Bragg reflection peak and color showed excellent uniformities in a relatively large area extending over several square centimeters (Supporting Information, Figure S3A). Note that the high uniformity of the CPC structure can be validated not only by the high frequencies of sharp reflection peaks in the transmission spectra but also by the visual appearance of the distinct Bragg reflection color. These results were reasonable considering the narrow diameter distribution of PNIPA hydrogel microparticles synthesized in this study. On the other hand, unlike the PNIPA hydrogel microparticles, uniform CPC structures could not be fabricated using monodisperse PS microparticles with the diameter of ∼190 nm. When monodisperse PS microparticles were sandwiched between two glass substrates, the CPCs showed irregular Bragg reflection peaks even in a 5 mm^2^ region (Supporting Information, Figure S3B). Therefore, the softness and elasticity of PNIPA hydrogel microparticles are expected to yield uniform and large-sized CPC structures in the aqueous suspension. Hereinafter, **PNIPA-5** was used to explore the photonic applications of PNIPA hydrogel microparticles, such as ion-sensing, temperature-sensing, and laser action.

### 3.4 Temperature-Responsive CPC Suspensions of PNIPA Hydrogel Microparticles

**Figure 4:**
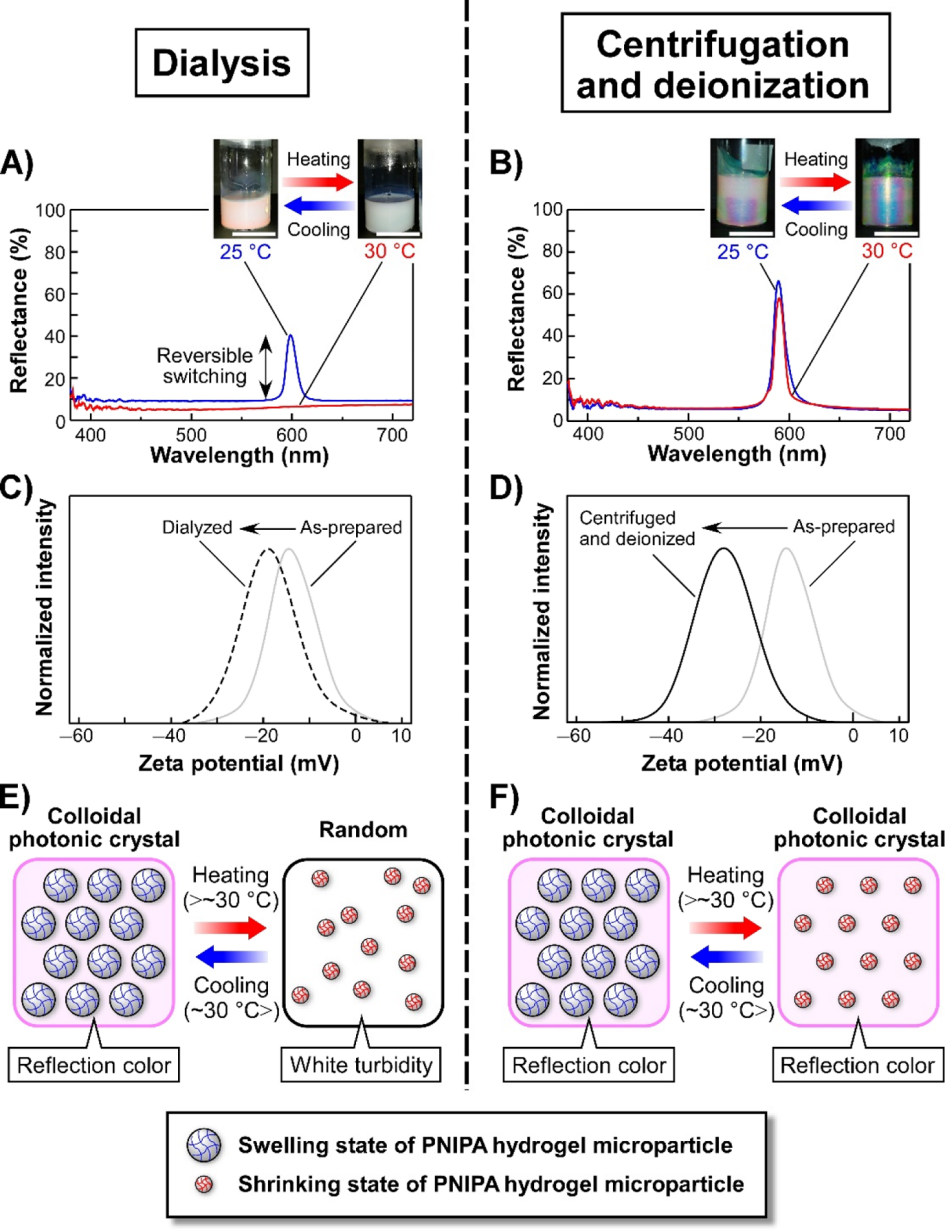
Comparative studies on the temperature-responsive reflection properties of CPC suspensions of **PNIPA-5** purified by dialysis (left-hand column) and the combination of centrifugation and deionization (right-hand column). (A,B) Reflection spectra and images of the CPC suspensions of **PNIPA-5** purified by dialysis (A) and by the combination of centrifugation and deionization (B) at 25 (blue lines) and 30 °C (red lines). The concentration of **PNIPA-5** in both cases was set to be 6 wt %. As can be watched on the demonstration video (Supporting Information, Movie S1), the dialyzed suspensions of **PNIPA-5** exhibited the dynamic and reversible switching in the visual appearance between the reflection color of red or green and white turbidity by changing the temperature. White scale bars in the images represent 10 mm. (C,D) Zeta potential profiles of the **PNIPA-5** suspensions purified by dialysis (C; dashed line) and by the combination of centrifugation and deionization (D; black solid line) at 25 °C. For comparison, the zeta potential profile of the as-prepared suspension of **PNIPA-5** is also shown in the figures (C,D; gray lines). (E,F) Schematic illustration of the proposed mechanism for the changes in structural arrangements of PNIPA hydrogel microparticles in the aqueous suspensions, which were purified by dialysis (E) and by the combination of centrifugation and deionization (F), induced by the temperature variation in the vicinity of 30 °C.

Moreover, the fluid suspensions of PNIPA hydrogel microparticles purified by dialysis exhibited not only visible reflection peaks in accordance to Bragg’s law but also reversible switching of the reflection peaks in response to the changes in temperature. When a 6 wt % dialyzed suspension of **PNIPA-5** was heated from 25 to 30 °C, the sharp reflection peak at ∼600 nm suddenly disappeared, with concomitant alteration in the visual appearance from a red reflection color to white turbidity (Figure 4A). These changes in the spectrum and color might originate from the changes in the structural arrangement of **PNIPA-5** from an ordered CPC state to a random state, which are triggered by the temperature-induced shrinkage of PNIPA hydrogel microparticles. Subsequently, when the **PNIPA-5** suspension was cooled to room temperature, the initial red reflection was immediately regained by the reassembly of **PNIPA-5** into the CPC structure, similar to the observations of two previous studies reported by the Lyon and Hu groups.^34,35^

Notably, we successfully demonstrated the rapid and dynamic color switching of the dialyzed suspensions of PNIPA hydrogel microparticles upon heating and cooling. As evident from the supporting video file (Supporting Information, Movie S1), the temperature-controlled color switching occurred for the CPC suspension just within the relative quick response times of ∼10 s during both heating and cooling processes. Such quick responses are presumably caused by the downsizing of the PNIPA hydrogel to several hundred nanometers.^32,33^

On the other hand, this type of temperature-controlled color switching was not observed for the aqueous suspensions of **PNIPA-5** purified by the combination of centrifugation and deionization. Although the centrifuged and deionized **PNIPA-5** suspension exhibited a reflection peak with a high intensity of 70% at ∼600 nm at 25 °C (Figure 4B, blue line), the reflection spectrum remained almost unchanged even after the heating of the suspension to over 30 °C (Figure 4B, red line). Indeed, there was no observable change in the reflection color before and after the heating at 30 °C (Figure 4B, images). This result is consistent with those of studies reported by the Asher and Okubo groups.^21,36^

The supporting video file (Supporting Information, Movie S2) also showed the clear differences in the reflection traits of CPC suspensions of **PNIPA-5** purified by dialysis and the combination of centrifugation and deionization. When small amounts of carbon black nanoparticles with the diameter of ∼16 nm were dispersed into these CPC suspensions in vials, the green reflection color was clearly observed due to the reduction of light scattering. As evident from this demonstration, the dialyzed CPC suspension exhibited the temperature-controlled switching between the reflection color and turbidity, while the reflection color of the centrifuged and deionized CPC suspensions was not changed at all even after heating to over 30 °C.

This difference between the Bragg reflection characteristics of CPC suspensions of **PNIPA-5** was characterized by the concentrations of ionic components in suspensions. The zeta potential profiles were measured for three kinds of **PNIPA-5** suspensions: as-prepared, after the purification by dialysis, and after the purification by the combination of centrifugation and deionization (Figure 4C,D). The as-prepared suspension was negatively charged with a zeta potential of −15 mV (Figure 4C,D, gray lines), whereas the suspension purified by dialysis showed a zeta potential of −20 mV (Figure 4C, dashed line). In contrast, when the suspension was purified by centrifugation and deionization, the zeta potential reached −28 mV, which was substantially lower than that of the suspension purified by dialysis (Figure 4D, black solid line). These experimental results indicated that the centrifuged and deionized **PNIPA-5** suspensions have relatively strong electrostatic repulsion forces, which are sufficiently high to retain the CPC structures in the aqueous suspensions. Furthermore, the zeta potentials were almost constant with the change in the temperature. For instance, the dialyzed **PNIPA-5** showed a constant value of zeta potential of around −20 mV at the temperature range between 25 and 40 °C (Supporting Information, Figure S4). The result implied the stable dispersion of PNIPA hydrogel microparticles in water even at the shrunken state after heating to 40 °C.

Based on the overall experimental results, Figure 4E,F illustrates the different mechanisms for the reflection properties of CPC suspensions of PNIPA hydrogel microparticles, which were purified by dialysis and the combination of centrifugation with deionization, respectively. In the case of the dialyzed CPC suspensions, the reflection color could be switched to white turbidity by heating these suspensions from 25 to 30 °C. As shown in Figure 1B, when the suspensions were heated to 30 °C, the diameter of PNIPA hydrogel microparticles reduced by ∼15% compared to that at 20 °C, leading to rapid deterioration of the CPC structures owing to the shrinkage of PNIPA hydrogel microparticles (Figure 4E, right illustration). In this state, the suspensions turned white and turbid because of the disassembly of CPCs by the random diffusion of shrunken PNIPA microparticles. When the suspensions were cooled to room temperature, the initial Bragg reflection colors were regained by the reassembly into uniform CPCs owing to the swelling of the PNIPA hydrogel microparticles (Figure 4E). Due to the temperature-controlled changes in the structural arrangement of PNIPA hydrogel microparticles, the dialyzed CPC suspensions showed the on-demand color switching between the Bragg reflection and white turbidity according to the temperature variation.

Contrary to the cases of the dialyzed PNIPA microparticles, the centrifuged and deionized CPC suspensions exhibited no change in the Bragg reflection colors even after these suspensions were heated over 30 °C (Figure 4F). In other words, the aqueous suspensions of PNIPA hydrogel microparticles preserved the reflection colors of CPC structures even at the shrunken state of microparticles after the heating treatment (Figure 4F, right illustration). When the centrifuged and deionized suspensions were heated to ∼30 °C, the diameters of PNIPA hydrogel microparticles decreased in the same manner as those in the dialyzed suspensions. Nevertheless, the PNIPA hydrogel microparticles had inherent capabilities to preserve the CPC structures owing to their strong electrostatic repulsion forces (Figure 4F, right illustration). As a result, the reflection peak wavelengths were unchanged before and after the heating because of no change in the lattice spaces of CPC structures, corresponding to *d*_*hkl*_ in eq 1. Therefore, the reflection colors of CPC suspensions remained constant even upon varying the temperature.

From the experimental results, we envisaged that the key factor of temperature-responsive reflection properties is the concentration of ionic components in the aqueous suspension of PNIPA hydrogel microparticles. Subsequently, the concentrations of K^+^, Na^+^, and SO_4_^2–^ were measured in two kinds of **PNIPA-5** suspensions purified by dialysis and the combination of centrifugation and deionization. The ionic components of K^+^ and SO_4_^2–^ might be derived from the decomposition of the thermal polymerization initiator of KPS used in the emulsion polymerization, and Na^+^ might be generated from the SDS surfactant. Herein, two purified **PNIPA-5** suspensions were quantitatively analyzed using the capillary electrophoresis. It was identified that the concentrations of all ionic components in the dialyzed **PNIPA-5** suspension are approximately 4–11 times higher than those in the centrifuged and deionized **PNIPA-5** suspensions (Supporting Information, Table S2). Accordingly, these ionic components at excessively high concentrations in suspensions diminished the electrostatic repulsion forces of PNIPA hydrogel microparticles to induce the disassembly of CPC structures after the heating treatment of the suspensions (Figure 4E). Therefore, it was surmised that the concentrations of ionic components in the CPC suspension is of prime importance to the temperature-induced color switching between the Bragg reflection and white turbidity.

**Figure 5:**
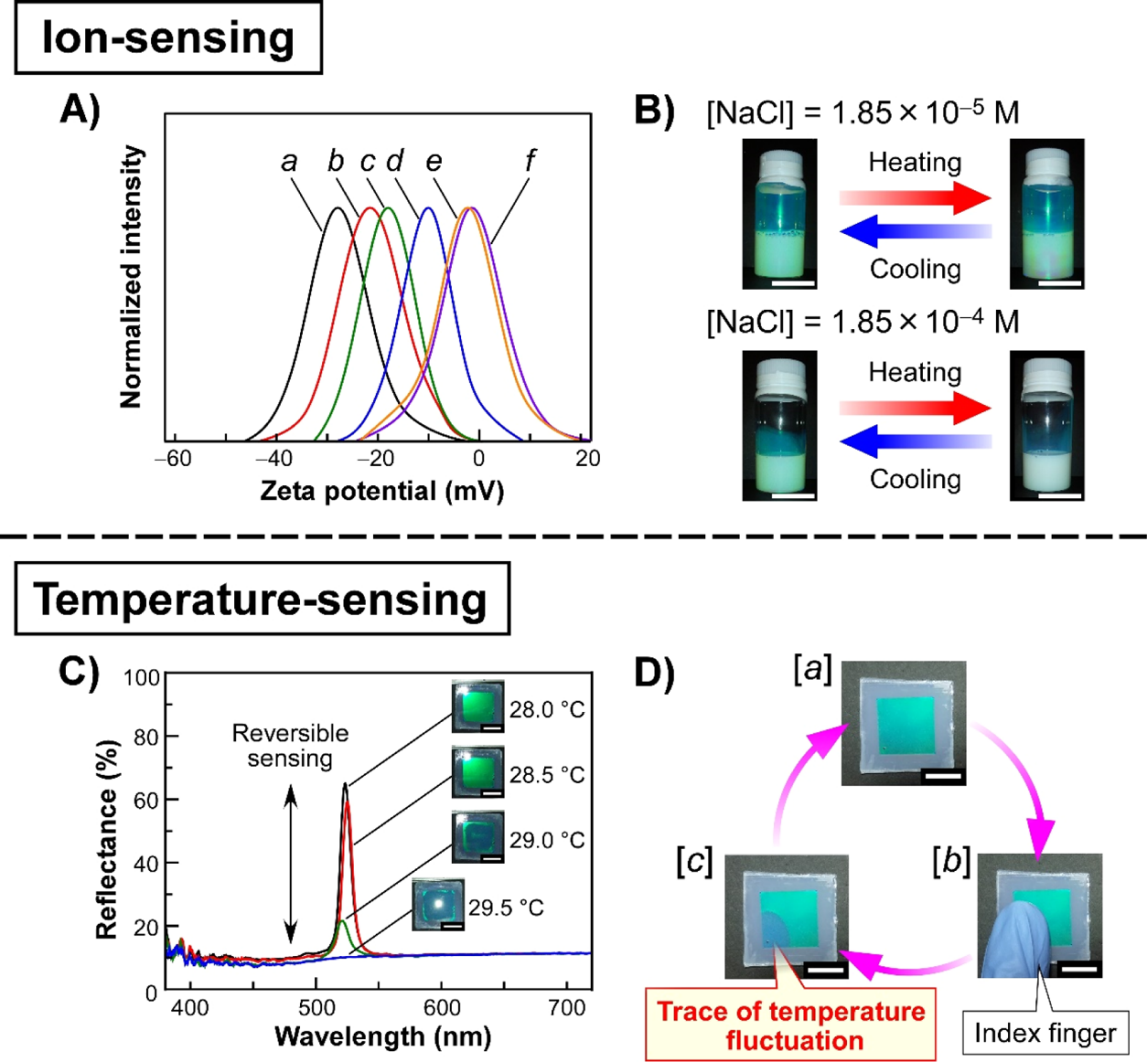
Ion sensing (upper panel) and temperature sensing (lower panel) using a CPC suspension of **PNIPA-5**. (A) Zeta potential profiles of **PNIPA-5** suspensions, purified beforehand by the combination of centrifugation and deionization, after the addition of NaCl at the following concentrations: 0 (curve *a*), 1.85 × 10^–6^ (curve *b*), 1.85 × 10^–5^ (curve *c*), 1.85 × 10^–4^ (curve *d*), 1.85 × 10^–3^ (curve *e*), and 1.85 × 10^–2^ M (curve *f*). (B) Temperature-induced switching of the reflection colors of 10 wt % suspensions of **PNIPA-5** added with NaCl at 1.85 × 10^–5^ (upper panel) and 1.85 × 10^–4^ M (lower panel) at the temperatures below (left-hand photographs) and above ∼30 °C (right-hand photographs). White scale bars in the image denote 10 mm. (C) Changes in the reflection spectrum of a 10 wt % CPC suspension of **PNIPA-5**, which was sandwiched between two glass substrates, at temperatures in the range from 28.0 to 29.5 °C. Insets show the corresponding reflection images, and white scale bars are 10 mm. (D) Demonstration of temperature sensing of the index finger of a human hand using the CPC suspension of **PNIPA-5**. Cyclic repetition of the following process was performed: the initial state (image *a*), touching the substrate surface with the index finger (image *b*), and successively detaching the finger from the substrate (image *c*). White scale bars represent 10 mm.

To prove the previously mentioned hypothesis, sodium chloride (NaCl) as an ionic substance was added into the centrifuged and deionized **PNIPA-5** suspensions. The corresponding results are shown in Figure 5A,B. In this experiment, the **PNIPA-5** concentration was adjusted to 10 wt %. Although the centrifuged and deionized suspension of **PNIPA-5** showed a zeta potential of −28 mV, the negative zeta potential values incrementally approached approximately −2 mV as the NaCl concentration was stepwise increased from 1.85 × 10^–6^ to 1.85 × 10^–2^ M (Figure 5A). Subsequently, we examined the reflection features of CPC suspensions of **PNIPA-5** before and after heating at 30 °C. When NaCl was added into the CPC suspension of **PNIPA-5** at the concentration of 1.85 × 10^–5^ M, the green Bragg reflection color was unchanged before and after the heating treatment (Figure 5B, upper images). In sharp contrast, surprisingly, the addition of 1.85 × 10^–4^ M NaCl into the CPC suspension enabled the reversible color switching between the green reflection and white turbidity, which was achieved by varying the temperature in the vicinity of 30 °C (Figure 5B, lower images). Such temperature-induced color switching happened because the electrostatic repulsion forces of PNIPA microparticles were weakened by the ionic components of Na^+^ and Cl^–^ dissolved in suspensions. This finding is substantially identical to those obtained for the **PNIPA-5** suspensions purified by dialysis (Figure 4A). Interestingly, the feature of the temperature-induced switching of the Bragg reflection was also observed for the CPC suspensions of **PNIPA-5** added with not only NaCl but also SDS, hydrogen chloride, and sodium hydroxide when their concentrations were adjusted over 1.85 × 10^–4^ M. It was found that critical concentrations of all the ionic substances to generate the temperature-induced switching of reflection colors are almost constant. Therefore, we grasped the mechanism in the temperature-induced color switching between the Bragg reflection and white turbidity using PNIPA hydrogel microparticles and that the concentrations of ionic components in the CPC suspension are an important factor in the preparation of active CPC suspensions with temperature-responsive reflection properties. Based on our overall experimental results, we have concluded that the temperature-induced switching of the Bragg reflection in CPC suspensions of PNIPA hydrogel microparticles reported in the previous studies by the Lyon and Hu groups can be reasonably explained by the existence of excessive ionic components in the CPC suspensions.^34,35^ In addition, it should be emphasized that the CPC suspensions of PNIPA hydrogel microparticles can be used in ion-sensing applications for the highly selective detection of negligible amounts of ionic components dissolved in water, as expressed through the disappearance of Bragg reflection colors due to temperature variation.

As an extension of our findings, we successfully demonstrated the temperature-sensing applications of the CPC suspensions of dialyzed **PNIPA-5**, as shown in Figure 5C,D. In this experiment, a 10 wt % CPC suspension of **PNIPA-5** was sandwiched between two glass substrates with a gap of ∼100 μm, and this sample was placed on a black background. When the temperature was adjusted to 28.0 °C, the CPC suspension exhibited a sharp reflection peak at 520 nm, and a green reflection color was observed (Figure 5C). Successive experiments confirmed the changes in the reflection spectrum of the CPC suspension upon elevating the temperature from 28.0 °C stepwise in intervals of 0.5 °C. The reflection peak gradually degraded upon heating the suspension in a narrow temperature range of ∼1.5 °C and eventually disappeared at 29.5 °C due to the temperature-induced deterioration of the CPC structure (Figure 5C, blue line). The suspension simultaneously altered from green reflection to light scattering (Figure 5C, insets). These changes in the reflection spectrum and color were reversible.

By utilizing the color switching behavior, the CPC suspension could be applied to the facile visualization of the human body temperature (Figure 5D). When we lightly touched the glass substrate containing the CPC suspension of **PNIPA-5** with the index finger, the green reflection color immediately disappeared because of the temperature-induced deterioration of the CPC structure by the transmission of the body temperature through the substrate. Subsequently, when the index finger was removed from the substrate, the green reflection color gradually recovered by the reassembly of the CPC structure of **PNIPA-5**. In this way, our findings would be significantly advantageous for the temperature-sensing applications requiring the distinction of imperceptible temperature fluctuations just near 28 °C through temporary color alterations.

### 3.5 Laser Action from CPC Suspensions of PNIPA Hydrogel Microparticles

**Figure 6:**
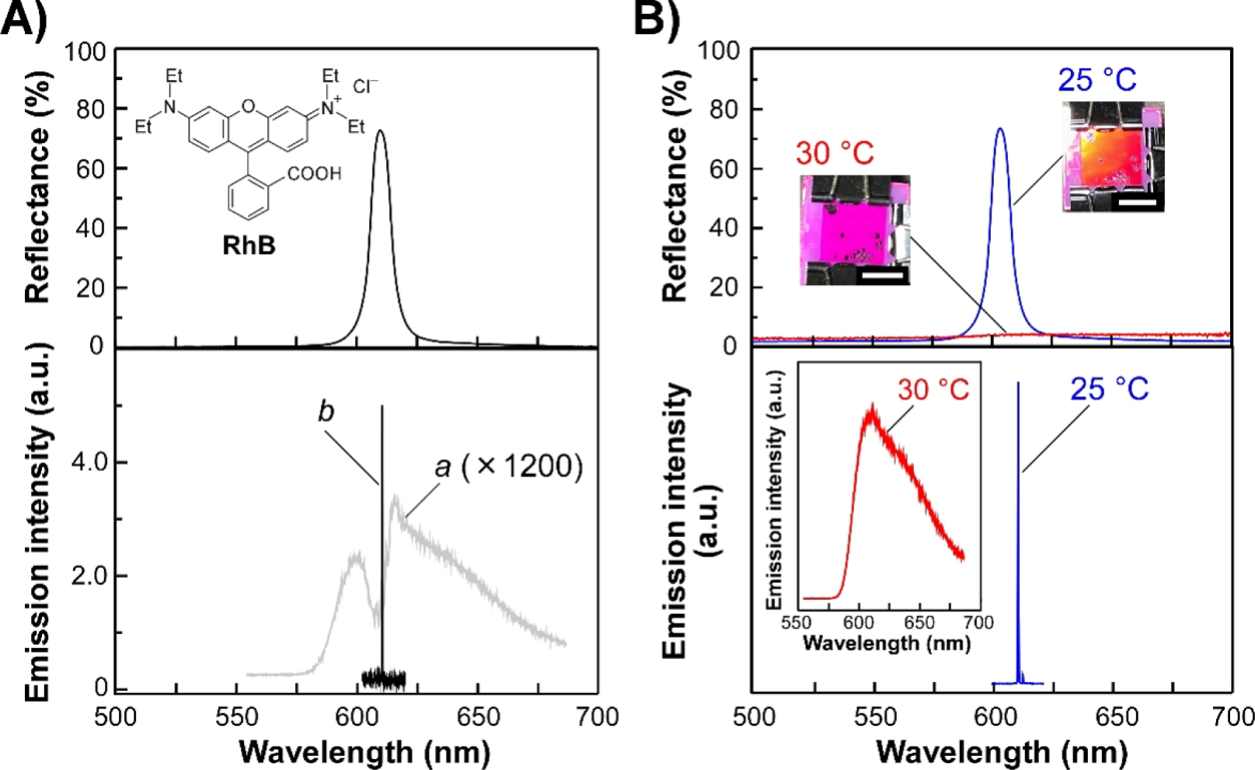
Optically excited laser action and its temperature-induced switching for the CPC suspension of **PNIPA-5**. (A) Reflection (upper panel) and emission (lower panel) spectra of the aqueous suspension of **PNIPA-5** with **RhB** at 25 °C. The inset of the upper panel shows the chemical structure of **RhB**. The **PNIPA-5** concentration was adjusted to 5.5 wt %. Emission spectra were measured by an excitation light at 532 nm with the energies of 140 (spectrum *a*) and 250 nJ pulse^–1^ (spectrum *b*). (B) Temperature-induced switching of the reflection (upper panel) and emission (lower panel) spectra of the suspension of **PNIPA-5** with **RhB** at 25 (blue lines) and 30 °C (red lines). Insets of the upper panel denote the photographs of the CPC suspension with **RhB** at 25 and 30 °C, and white scale bars represent 10 mm. As shown in the inset of the lower panel, the emission spectrum was considerably changed from the sharp laser peak to the broad spontaneous emission band when the suspension was heated above ∼30 °C.

Exploiting the peculiar property of PNIPA hydrogel microparticles—that visible Bragg reflection and its high reflectance are observed for the aqueous CPC suspensions—we then developed another new potential application of PNIPA hydrogel microparticles as PCs for the generation of the stimulated emission based on the PBG effect of CPC suspensions. At first, in order to attain the laser action, 0.5 wt % of a water-soluble light-emitting dye of **RhB** (Figure 6A, upper panel, inset) was dissolved into a 5.5 wt % CPC suspension of **PNIPA-5**. The reflection spectrum of the CPC suspension showed a sharp and intense Bragg reflection band at 610 nm (Figure 6A, upper panel), which completely overlapped with a broad spontaneous emission (fluorescence) band of **RhB** with the maximum peak at 610 nm. Considering that the optical gain spectrum is generally similar to the spontaneous emission spectrum, it was expected that the optically excited laser action is efficiently generated using a 5.5 wt % CPC suspension of **PNIPA-5** with **RhB**.

Emission properties of the CPC suspension of **PNIPA-5** containing **RhB** were studied by optical excitation with an incident light at 532 nm from a *Q*-switched Nd:YAG laser beam combined with OPO, as mentioned in Section 2.5. Herein, the laser experiments were performed at 25 °C. At a relatively low excitation energy of up to 140 nJ pulse^–1^, the emission intensity proportionally increased in line with the excitation energy. Optical excitation at 140 nJ pulse^–1^ resulted in the partial inhibition of the emission intensity in the wavelength range of 600–615 nm in the broad spontaneous emission spectrum of **RhB** (Figure 6A, lower panel, spectrum *a*). At this stage, the emission intensity of **RhB** was inhibited within the reflection band of the CPC suspension, arising from the confinement of photons emitted from **RhB** within the PBG wavelength range of the CPC suspension. When the excitation energy was increased to 250 nJ pulse^–1^, the emission intensity was enhanced by 3 or more orders of magnitude greater, accompanied by the abrupt spectral narrowing from 74 to 0.14 nm (Figure 6A, lower panel, spectrum *b*). Thus, this emission phenomenon is regarded as the laser action. A single laser peak emerged near the longer-wavelength edge of the Bragg reflection band at 610 nm by the PBG effect. Thus, the laser peak appeared by the strong localization and amplification of the photons emitted from **RhB** near the longer-wavelength edge of the reflection band of the uniform CPC suspension. The emission spectral linewidth (Δλ) in this laser peak was as narrow as 0.14 nm. In general, the quality factor (*Q*) of the CPC laser microcavity can be expressed as
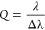
3where λ corresponds to the wavelength of the laser peak. From this laser spectrum, the *Q* value of the CPC laser microcavity was estimated to be 4.4 × 10^3^ (Figure 6A, lower panel, spectrum *b*). The *Q* value recorded herein was reasonably high compared to those of the laser microcavities fabricated using organic and polymer materials.^37−46,54−58^ Such a sharp laser peak is of technological advantage in the fabrication of high-density photonic devices.

In order to find a threshold excitation peak power in this laser action, further experiments revealed the changes in the emission intensity at the laser peak wavelength of 610 nm and the corresponding spectral linewidth upon the increasing optical excitation energy (Supporting Information, Figure S5). Consequently, the threshold excitation energy needed to generate the laser feedback effect was found to be 150 nJ pulse^–1^. In this experiment, the pulse duration was set to ∼6 ns, and the focused diameter of excitation beam was 40 μm. Therefore, the threshold peak power for the laser action was estimated to be 2.0 MW cm^–2^. To the best of our knowledge, this threshold value is satisfactorily low as compared to the previously reported values for the laser action from other laser microcavities fabricated using organic and polymer materials.^37−46,54−58^ Importantly, a stable laser action was observed during the experiments, stemming from the low-threshold optical excitation. Accordingly, there was no damage of the CPC suspensions, caused by the laser ablation or temperature-induced volume changes of PNIPA microparticles, after the laser experiments. Such a low-threshold laser action with a single and narrow peak originated from the uniform CPC suspensions with highly efficient Bragg reflection, acting as the PBG in the CPC laser microcavity.

Reminding the intrinsic features of temperature-responsive PNIPA microparticles that the reflection band is reversibly switchable with temperature (Figures 4A and 5C), we then sought to demonstrate the control of the laser action from the CPC suspension of **PNIPA-5** with **RhB** by changing the temperature (Figure 6B). Although the CPC suspension of **PNIPA-5** with **RhB** showed a reflection band at ∼610 nm at 25 °C, the subsequent heating of the suspension at 30 °C led to the complete disappearance of this reflection band due to the disassembly of the CPC structure caused by the temperature-induced shrinkage of **PNIPA-5** (Figure 6B, upper panel). Following this reflection spectral change, the red reflection color vanished after heating from 25 to 30 °C (Figure 6B, upper panel, insets). Although the optically excited laser action was constantly observed at 25 °C, the laser peak was synchronously switched with the changes in the reflection properties of CPC suspensions (Figure 6B, lower panel). When the temperature was tuned from 25 to 30 °C, the emission spectrum was immediately changed from the laser peak to the spontaneous emission band, whereupon the laser action could not be generated at all (Figure 6B, lower panel). This response can be attributed to the disassembly of the CPC structure triggered by the shrinkage of **PNIPA-5**, which was also evidenced from not only the lack of the reflection peak (Figure 6B, upper panel, red line) but also no partial inhibition in the emission spectrum by the reflection band (Figure 6B, lower panel, inset). When this suspension was cooled to 25 °C, the reflection band and laser peak were reversibly recovered. This temperature-responsive laser action can also be explained based on our empirical findings that the temperature-induced switching of CPC structures is governed by the addition of ionic substances, including **RhB**, into the suspensions of **PNIPA-5**. The results are considered as a breakthrough for the fabrication of miniaturized laser devices with the temperature-induced switching, which are utilized by the PBG effect of aqueous CPC suspensions including temperature-responsive microparticles of polymer hydrogels.

### 3.6 Reusability of PNIPA Hydrogel Microparticles as Laser Microcavities

**Figure 7:**
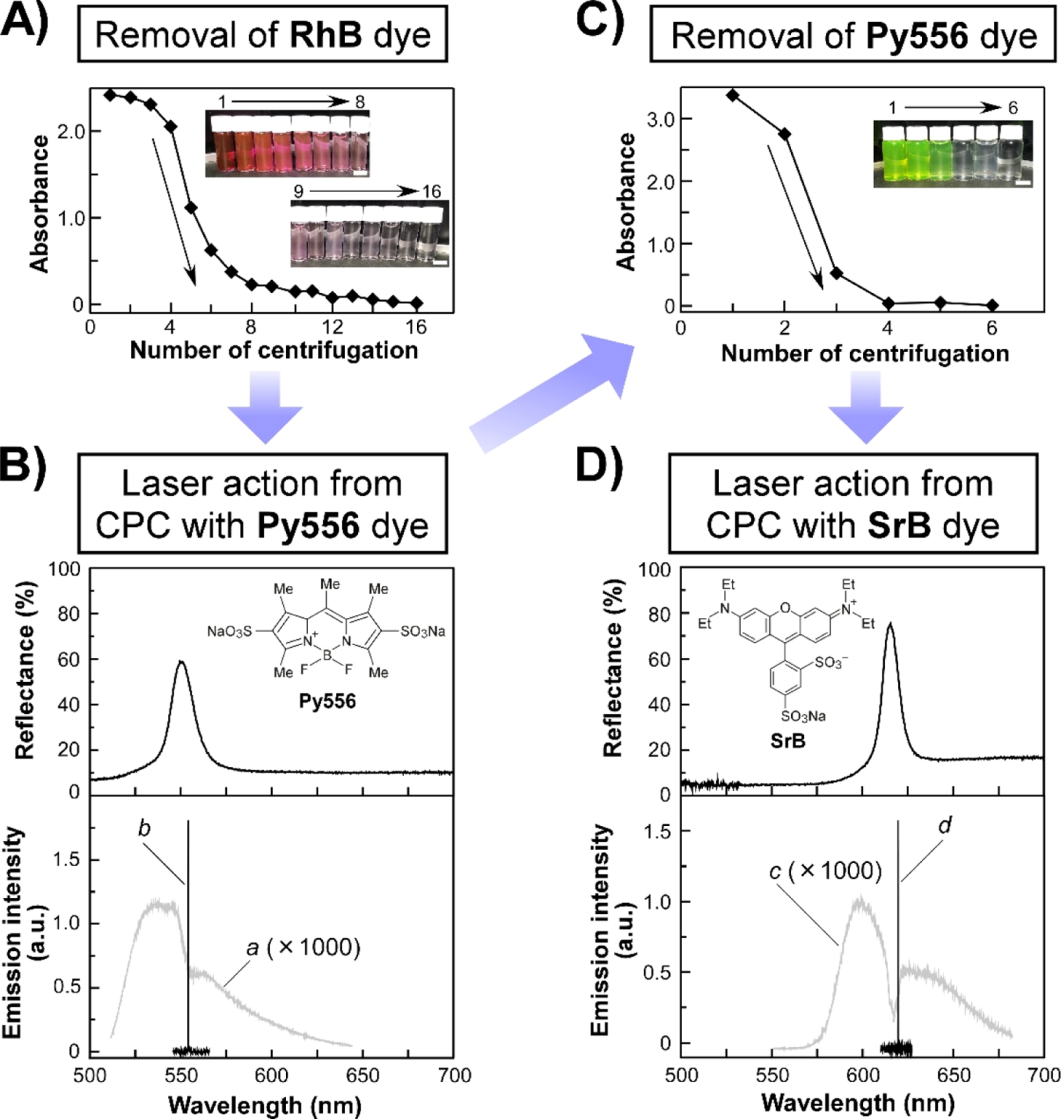
Reuse demonstration of **PNIPA-5** as CPC laser microcavities to realize laser action at other wavelengths by using different dyes and tuning the reflection band of the CPC suspension. A course of experiments shown in (A–D) were consecutively performed using an aqueous CPC suspension of **PNIPA-5** with **RhB** used in Figure 6 as the starting sample. (A) Changes in the absorbance of **RhB** at 560 nm in the supernatant after the centrifugation of the suspension of **PNIPA-5** with **RhB**, used in Figure 6, for up to 16 rounds. Insets show the images of the aqueous supernatant obtained after repeated centrifugation processes, and white scale bars in the images mean 10 mm. (B) Reflection (upper panel) and emission (lower panel) spectra of the CPC suspension of **PNIPA-5**, purified beforehand as indicated in (A), containing **Py556**. The inset of the upper panel shows the chemical structure of **Py556**. The **PNIPA-5** concentration was adjusted to 7 wt %. Emission spectra were measured by an excitation light at 500 nm with the energies of 110 (spectrum *a*) and 180 nJ pulse^–1^ (spectrum *b*). (C) Changes in the absorbance of **Py556** at 490 nm in the aqueous supernatant after the centrifugation of the suspension of **PNIPA-5** with **Py556**, used in (B), for up to 6 rounds. The inset represents the images of the aqueous supernatant acquired after the centrifugation, and the white scale bar in the image is 10 mm. (D) Reflection (upper panel) and emission (lower panel) spectra of the CPC suspension of **PNIPA-5**, purified beforehand as indicated in (C), containing **SrB**. The inset of the upper panel depicts the chemical structure of **SrB**. The **PNIPA-5** concentration was adjusted to 5 wt %. Emission spectra were measured by an excitation light at 532 nm with the energies of 90 (spectrum *c*) and 120 nJ pulse^–1^ (spectrum *d*).

Finally, we substantiated the reuse of PNIPA microparticles as the CPC laser microcavities to generate the optically excited laser action at other wavelengths using different light-emitting dyes. Figure 7 shows one of the most essential results of this study. The aqueous CPC suspension of **PNIPA-5** with **RhB**, as used in Figure 6, was consecutively adopted for a course of experiments to reuse **PNIPA-5** (Figure 7A–D).

At first, the suspension of **PNIPA-5** with **RhB** was separated into two layers by centrifugation: an aqueous supernatant containing **RhB** in the upper layer and an aggregation of **PNIPA-5** flocculated at the bottom of the centrifuge tube. Following the careful removal of the supernatant from the tube, the absorption spectrum was acquired using a spectrophotometer to confirm the **RhB** remaining in the supernatant. Figure 7A shows the changes in the absorbance of **RhB** at the maximum absorption wavelength (λ_max_) of 560 nm in the supernatant during 16 rounds of centrifugation of the suspension of **PNIPA-5** with **RhB**. Although **RhB** was not completely removed from the suspension of **PNIPA-5** by the first-round centrifugation, the repeated centrifugation ensured the removal of **RhB**, as indicated by the gradual decrease in the absorbance of **RhB** from 2.5 to 0.01. After 16 rounds of centrifugation, the red color of the supernatant faded into optical transparency, as recognized by the naked eye (Figure 7A, insets). Consequently, almost all **RhB** molecules were eliminated from the suspension of **PNIPA-5**. Thus, the purified **PNIPA-5** was collected again. Indeed, DLS measurements revealed that the diameter and its CV of the purified **PNIPA-5** at 20 °C are 258 nm and 9.8, respectively. These values are almost identical to those before the reuse experiment (Supporting Information, Figure S6). The purified **PNIPA-5** showed no structural damage even after 16 rounds of centrifugation and could be well-dispersed in water without their aggregations. This finding motivated us to examine the reusability of purified **PNIPA-5** for the laser action at other wavelengths by dissolving different light-emitting dyes in the **PNIPA-5** suspension.

As the successive trial, **Py556** (Figure 7B, upper panel, inset) with the maximum spontaneous emission wavelength of 530 nm was used as a light-emitting dye. Figure 7B shows the reflection and emission spectra of the CPC suspension of **PNIPA-5** with **Py556**. Similar to the demonstration in the preceding experiment, the reflection band of the CPC suspension could be on-demand controlled by adjusting the concentration of **PNIPA-5** (Figure 2). Because of the rational fine tuning of the reflection band by the **PNIPA-5** concentration, a 7 wt % CPC suspension of **PNIPA-5** showed a reflection band at 550 nm (Figure 7B, upper panel), which overlapped with the spontaneous emission band of **Py556**. Subsequently, the CPC suspension containing 0.3 wt % of **Py556** was optically excited with an incident light at 500 nm. With the increase in the excitation energy from 110 to 180 nJ pulse^–1^, the emission spectral shape was drastically changed from the broad spontaneous emission band of **Py556** with a partial inhibition at ∼550 nm to the single laser peak with a narrow spectral linewidth of 0.08 nm located near the longer-wavelength edge of the reflection band of 553 nm (Figure 7B, lower panel). At this stage, the *Q* value were estimated to be 6.9 × 10^3^. This laser action also indicated that the reflection band of the CPC suspension acts as the PBG in the CPC laser microcavity, as explained in the preceding section. Thus, the single laser peak could be tuned from 610 to 555 nm not only by changing from **RhB** to **Py556** but also by adjusting the reflection band of the CPC suspension from 610 to 550 nm in relation to the **PNIPA-5** concentration.

Finally, the possibility of the laser action at 620 nm was examined by removing **Py556** from the CPC suspension, followed by dissolving **SrB** (Figure 7D, upper panel, inset) as a light-emitting dye into the CPC suspension of purified **PNIPA-5**. After repeated centrifugation cycles of the suspension used in Figure 7B, the absorbance of **Py556** at 490 nm in the supernatant undermined from 3.2 to 0.01 after 6 rounds of centrifugation (Figure 7C). Although the supernatant showed the green color of **Py556** after the first round of centrifugation, the gradual decolorization was observed upon continuous centrifugation (Figure 7C, inset). At this time, **PNIPA-5** was finely purified by the repeated centrifugation, as confirmed by DLS measurements (Supporting Information, Figure S6). Subsequently, the concentration of purified **PNIPA-5** in water was adjusted to 5 wt %, thereby resulting in the emergence of a reflection band of the CPC suspension at 615 nm (Figure 7D, upper panel). After that, another light-emitting dye of **SrB** (Figure 7D, upper panel, inset) was dissolved at the concentration of 0.3 wt % into the CPC suspension of **PNIPA-5**. Similar to the cases of light-emitting dyes of **RhB** (Figure 6A) and **Py556** (Figure 7B), the single laser peak was also observed at 620 nm for the CPC suspension with **SrB** by optical excitation with an incident light at 532 nm. When the excitation energy was increased from 90 to 120 nJ pulse^–1^, the broad emission band of **SrB** was changed to the single laser peak at 620 nm, almost corresponding to the longer-wavelength edge of the reflection band of the CPC suspension (Figure 7D, lower panel). For this laser action, the high-resolution emission spectral measurement revealed a narrow spectral linewidth of 0.40 nm, corresponding to the *Q* value of 1.6 × 10^3^.

The overall results highlighted that our PNIPA hydrogel microparticles can be repeatedly used as CPC laser microcavities for the facile exchange of the laser peak wavelength not only by choosing the light-emitting dye but also by adjusting the concentration of PNIPA hydrogel microparticles (Figure 7). Moreover, the laser peak can be switched through the self-assembly/disassembly events of the CPC suspension of PNIPA microparticles in response to temperature (Figure 6B). Thus, these purifiable and reusable materials demonstrated herein provide promising clues for the conservation of the precious synthetic materials produced from finite petroleum resources by their continual reuse for the realization of a sustainable society.

## 4 Conclusions

In this study, we have established promising strategies to prepare not only monodisperse PNIPA hydrogel microparticles with diameters of several hundred nanometers but also CPC suspensions of these PNIPA hydrogel microparticles with visible Bragg reflection for the sensor and laser applications. The emulsion polymerization of PNIPA hydrogel precursors with an SDS surfactant facilitated the preparation of monodisperse PNIPA hydrogel microparticles, whose diameters could be finely controlled by adjusting the SDS concentration during the polymerization. These PNIPA hydrogel microparticles showed the self-organization of uniform CPC structures with a relatively high reflectance of ∼60% in the visible wavelength region, even in fluid suspensions. Interestingly, the addition of small amounts of ionic substances into the centrifuged and deionized CPC suspensions enabled the dynamic color switching between Bragg reflection and white turbidity in response to changes in temperature. These reversible color changes originated from the self-assembly/disassembly of CPC structures by changing the temperature, which involved the weakening of the electrostatic repulsion forces of PNIPA hydrogel microparticles by the ionic components in suspensions. Therefore, the concentrations of ionic components in the CPC suspensions were identified as a key factor leading to the temperature-controlled color switching, which is technologically important in temperature- and ion-sensing applications.

As a further advancement of our findings, we also developed a new potential use of the CPC suspensions of PNIPA hydrogel microparticles in the laser applications. Optical excitation of the CPC suspensions with light-emitting dyes led to a single and narrow laser peak near the longer-wavelength edge of the reflection band by the PBG effect of CPC suspensions. Furthermore, the light-emitting dyes were readily removed from the CPC suspensions by centrifugation. We successfully demonstrated the reuse of PNIPA hydrogel microparticles as CPC laser microcavities to generate the laser action at other wavelengths. This was accomplished by not only changing the light-emitting dye but also adjusting the reflection band of CPC suspensions by the microparticle concentration. Thus, these hydrogel microparticles could be persistently reused by centrifugation, enabling the reduction of the excessive consumption of finite petroleum resources. This report would contribute to the establishment of new circular economy concepts using these purifiable and reusable materials for a sustainable society for future generations.

## Supporting Information Available

Experimental details for the characterization of physical properties; optimization for the synthesis condition of emulsion polymerization with PNIPA hydrogel precursors and size properties of the resultant PNIPA hydrogel microparticles; SEM images of PNIPA hydrogel microparticles synthesized at different concentration of SDS; mean diameters and their CVs of **PNIPA-5** in the temperature range between 20 and 60 °C; superimposed transmission spectra of CPC suspensions of PNIPA hydrogel and PS microparticles by the transmission spectral imaging measurement system using a hyperspectral camera; concentrations of K^+^, Na^+^, and SO_4_^2–^ in an aqueous suspension of **PNIPA-5** purified by dialysis or by centrifugation and deionization; zeta potential profiles of the aqueous suspension of **PNIPA-5**, purified by dialysis in advance, as a function of temperature ranging between 25 and 40 °C; changes in the emission intensity and emission spectral linewidth for the CPC suspension of **PNIPA-5** with **RhB** as a function of the excitation energy of the 532 nm light from an Nd:YAG laser beam; and size properties of **PNIPA-5** before and after the reuse experiments (PDF)
Color switching of dialyzed suspensions of PNIPA hydrogel microparticles between a reflection color of red or green and white turbidity upon heating and cooling processes (MP4)
Comparison of the visual appearance for two kinds of suspensions of PNIPA hydrogel microparticles, purified by dialysis or the combination of centrifugation and deionization, containing small amounts of carbon black nanoparticles upon heating and cooling processes (MP4)
