# Supplementary material for: Colloidal Photonic Crystals of Reusable Hydrogel Microparticles for Sensor and Laser Applications
Source: ACS Appl Mater Interfaces. 2021 Nov 25;13(48):57893–907. doi: 10.1021/acsami.1c16500 (PMC8662631; doi:10.1021/acsami.1c16500)
Supplement: Supplementary file 1 — am1c16500_si_001.pdf [file am1c16500_si_001.pdf]

Supporting Information

Colloidal Photonic Crystals of Reusable Hydrogel Microparticles for Sensor and Laser Applications

Naoto Iwata, Takeru Koike, Kaya Tokuhiro, Ryu Sato, and Seiichi Furumi*

Department of Applied Chemistry, Faculty of Science,
Tokyo University of Science,
1-3 Kagurazaka, Shinjuku, Tokyo 162-8601, Japan

*E-mail: furumi@rs.tus.ac.jp.

1. Experimental Details for Characterization of Physical Properties

Hydrodynamic diameters of poly(*N*-isopropylacrylamide) (PNIPA) hydrogel microparticles in ultrapure water were measured in the temperature range between 20 and 60 °C using a particle size analysis system based on the dynamic light scattering (DLS) technique (Nanotrac UPA-UT151, Nikkiso) connected with a temperature-controllable isothermal liquid circulator (NCB-1200, EYELA). Coefficients of variation (CV) in microparticle diameter, which are numerically defined as the ratio of the standard deviation to the average diameter, were calculated from the DLS results. Scanning electron microscopy (SEM) images of the dried PNIPA microparticles on silicon wafer substrates were taken on a field-emission SEM (JSM-7200F, JEOL) at an acceleration voltage of 10 kV. Before SEM observation, the surface of sample was deposited with a thin layer of platinum using a magnetron sputtering (MSP-1S, Vacuum Device). Zeta potential measurements were conducted using a particle charge analysis system (Zetasizer Nano ZS, Malvern). Concentration of cationic and anionic components in the suspensions of PNIPA hydrogel microparticles were quantitatively analyzed using a capillary electrophoresis system (Agilent 7100, Agilent Technologies).

For optical measurements of transmission and reflection spectra, the aqueous colloidal photonic crystal (CPC) suspension of PNIPA hydrogel microparticles was sandwiched between two glass substrates (~0.9 × 25 × 25 mm^3). The interior gap between the glass substrates was adjusted using polytetrafluoroethylene film spacers with the thickness of ~100 μm. Transmission spectra were measured at room temperature using an ultraviolet–visible–near-infrared spectrophotometer (UV Probe 3150, Shimadzu). Transmission spectral imaging measurements were conducted using an original system composed of a mirror scanning spectral measurement device (HSC-800, JFE Techno-Research Co. Ltd.) combined with a hyperspectral camera (ImSpector V8C, Specim). Reflection spectra were recorded using a compact charge-coupled device spectrometer (USB2000+, Ocean Optics) equipped with both a halogen light source (HL-2000, Ocean Optics) and an optical fiber with a reflection probe (R200-7-UV-VIS, Ocean Optics). When the white light as a probing beam was irradiated from the surface normal, the reflection spectra were taken in the direction of regular reflection. Reflection images were acquired at room temperature for the samples on a black background. The temperature of suspensions of PNIPA hydrogel microparticles was precisely controlled using a temperature controller (HS1, Mettler-Toledo) equipped with a hot stage (HS82, Mettler-Toledo). To improve the visibility of reflection colors, small amounts of carbon black nanoparticles with the diameter of ~16 nm (#950, Mitsubishi Chemical Co.) were dispersed in the aqueous CPC suspensions of PNIPA hydrogel microparticles.

2. Supporting Figures and Tables

A)

Sample code	Amount of SDS (g)	Amount of BIS (g)	Mean diameter of PNIPA microparticle (nm)	
			at 25 °C	at 45 °C
Sample a	0.10	3.0	319	217
Sample b	0.10	2.5	313	197
Sample c	0.10	2.0	307	190

B)

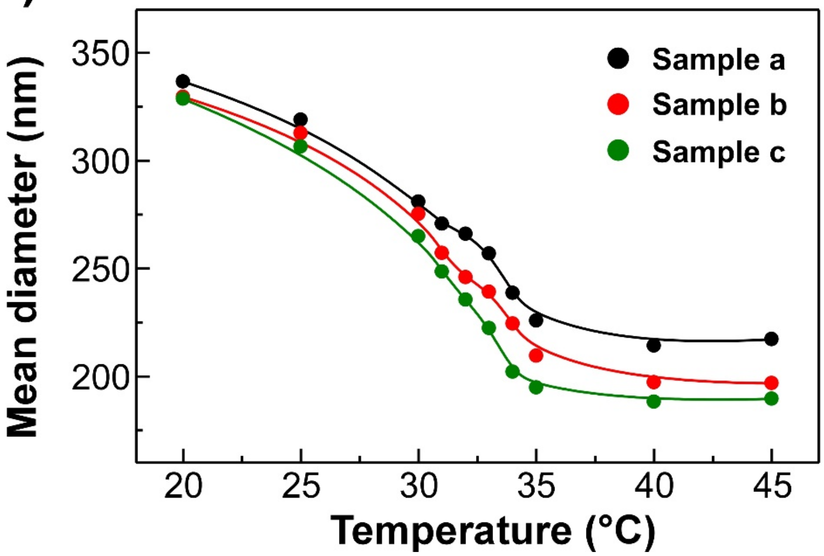

Figure S1. Optimization for the synthesis condition of emulsion polymerization with PNIPA hydrogel precursors and size properties of the resultant PNIPA hydrogel microparticles analyzed by the DLS measurements. A) List of amounts of SDS and BIS during the emulsion polymerization and mean diameters of the PNIPA hydrogel microparticles at 25 and 45 ºC. B) Changes in the mean diameters of PNIPA hydrogel microparticles of Sample a (black circles), b (red circles), and c (green circles) as a function of temperature.

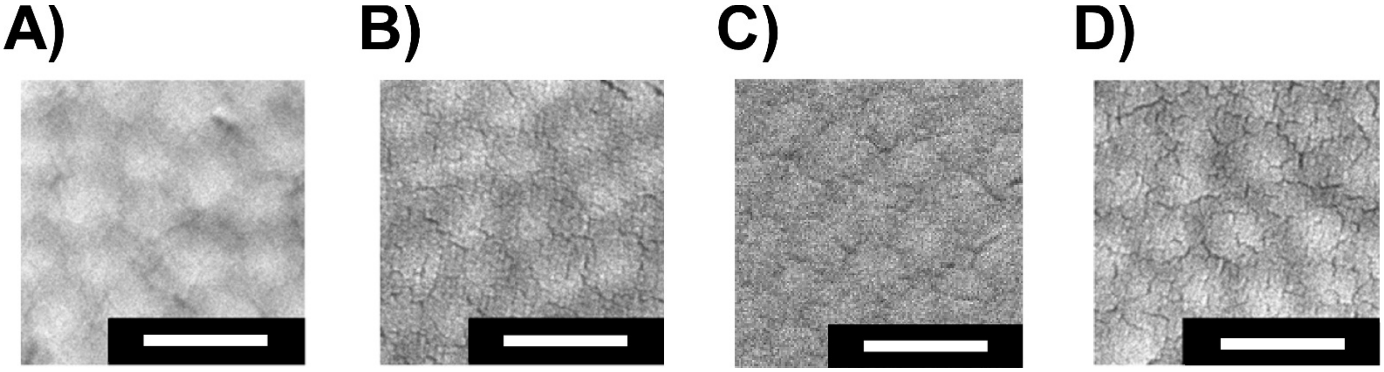

Figure S2. Representative SEM images of PNIPA hydrogel microparticles with different diameters, **PNIPA-4** (A), **PNIPA-5** (B), **PNIPA-6** (C), and **PNIPA-7** (D), collapsed on a silicon wafer substrate after drying. As mentioned in Table 1, the SDS concentrations in emulsion polymerization to prepare **PNIPA-4**, **PNIPA-5**, **PNIPA-6**, and **PNIPA-7** were adjusted in 0.87, 1.16, 1.44, and 1.73 mM, respectively. From the SEM images, the microparticle diameters of collapsed **PNIPA-4**, **PNIPA-5**, **PNIPA-6**, and **PNIPA-7** were estimated to be ~180, ~170, ~150, and ~140 nm, respectively. White scale bars represent 300 nm.

Table S1. Mean diameters and their CVs of **PNIPA-5** measured at the temperatures from 20 to 60 ºC.

Temperature (ºC)	Mean diameter (nm)[a)]	CV in diameter (%)[a)]
20	263	9.8
25	248	10.2
30	220	10.2
35	161	9.0
40	152	8.1
45	152	7.8
60	149	7.5

a) These values were obtained by the DLS measurements at respective temperatures.

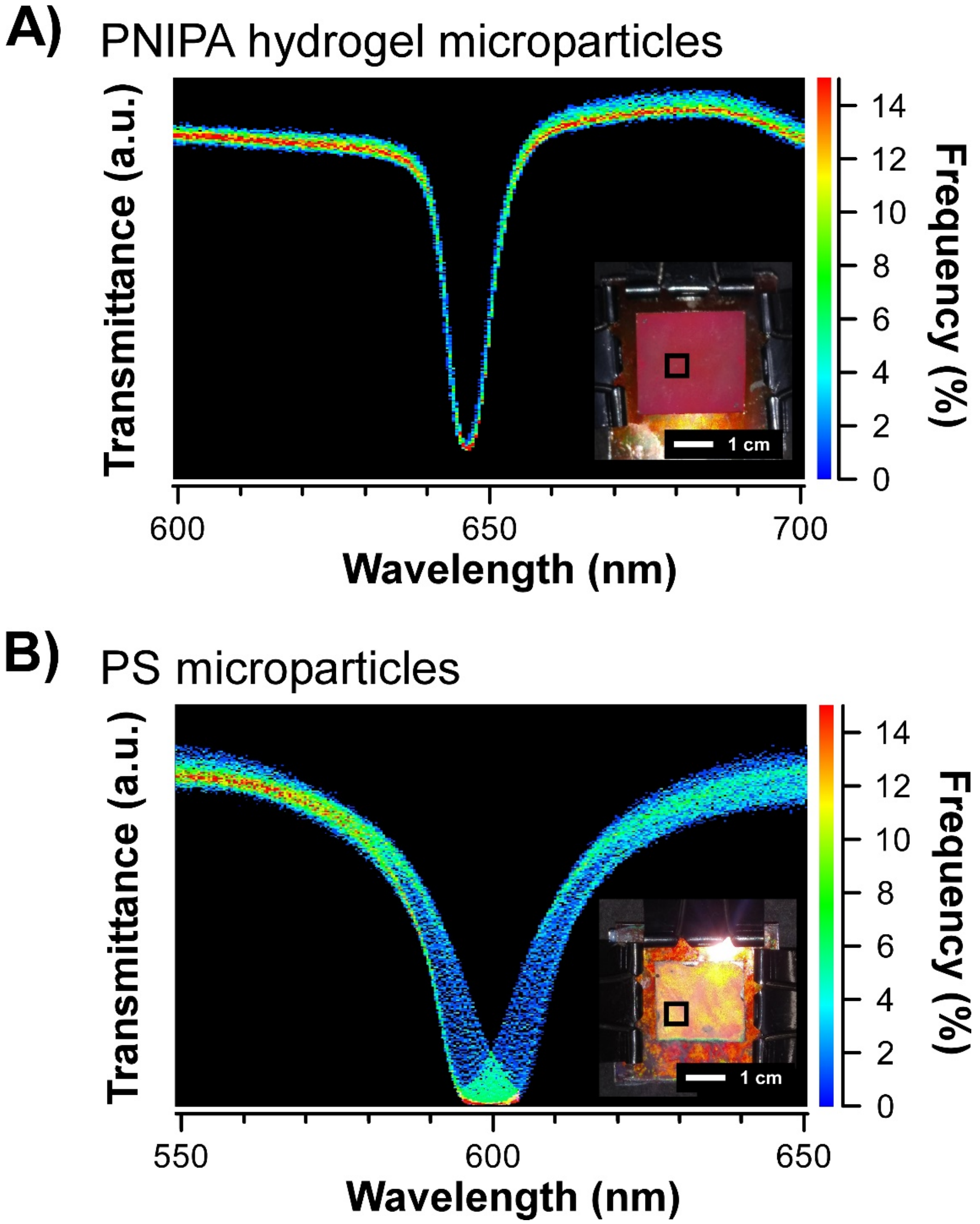

Figure S3. Superimposed transmission spectra of CPC suspensions of PNIPA hydrogel microparticles (A) and PS microparticles (B) sandwiched between two glass plates, which were acquired by the transmission spectral imaging measurement system using a hyperspectral camera according to the Experimental section. The monodisperse PS microparticles with the diameter of ~190 nm were synthesized by the emulsion polymerization of styrene monomer with an SDS surfactant. The insets show the photographs of CPC suspensions, and white scale bars represent 1 cm. The black squares are the measurement area of 25 mm^2. The in-plane resolution of spectral images was estimated to be 250 × 250 μm^2, because there were 400 pieces of transmission spectra in 25 mm^2.

Table S2. Concentrations of K^+, Na^+, and SO_4^{2-} in aqueous suspension of **PNIPA-5** purified by dialysis or centrifugation and deionization.

Concentrations of ionic component (mg L^{-1})	Purification procedure	
	Dialysis	Centrifugation and deionization
K^+	1.73	0.25
Na^+	4.84	1.10
SO_4^{2-}	0.44	0.04

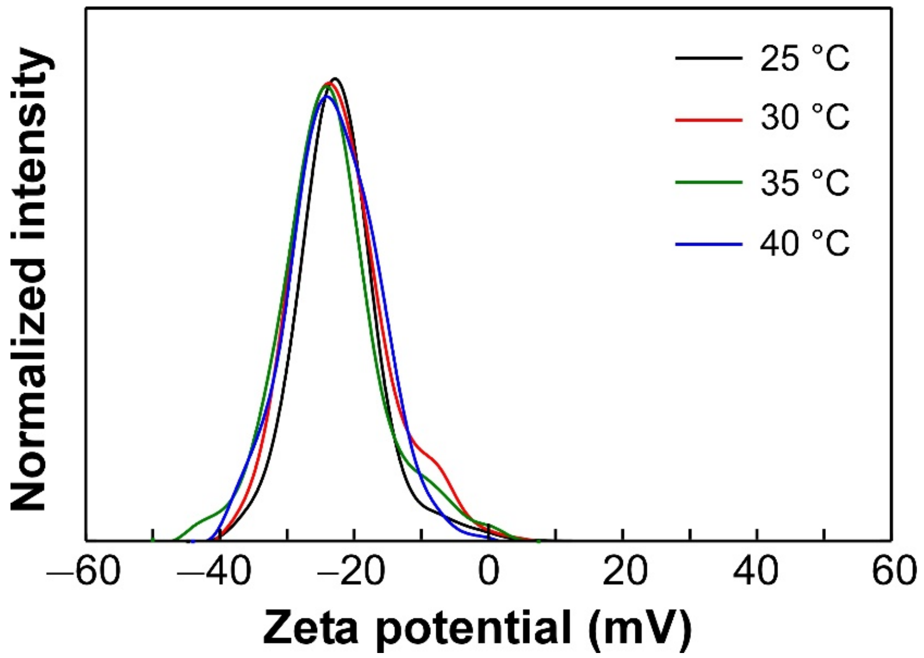

Figure S4. Zeta potential profiles of the aqueous suspension of **PNIPA-5**, purified by dialysis in advance, as a function of temperature between 25 and 40 ºC.

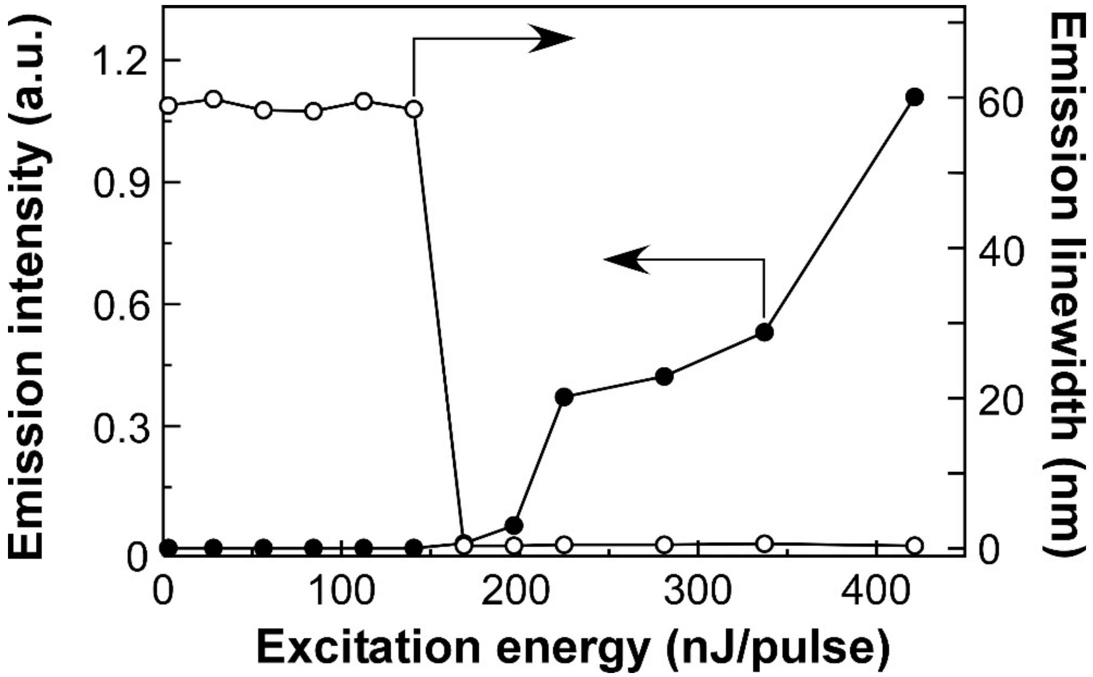

Figure S5. Changes in the emission intensity at the laser peak wavelength of 610 nm (closed circles) and emission spectral linewidth (open circles) for the CPC suspension of **PNIPA-5** with **RhB** as a function of excitation energy of the 532 nm light from an Nd:YAG laser beam.

A)

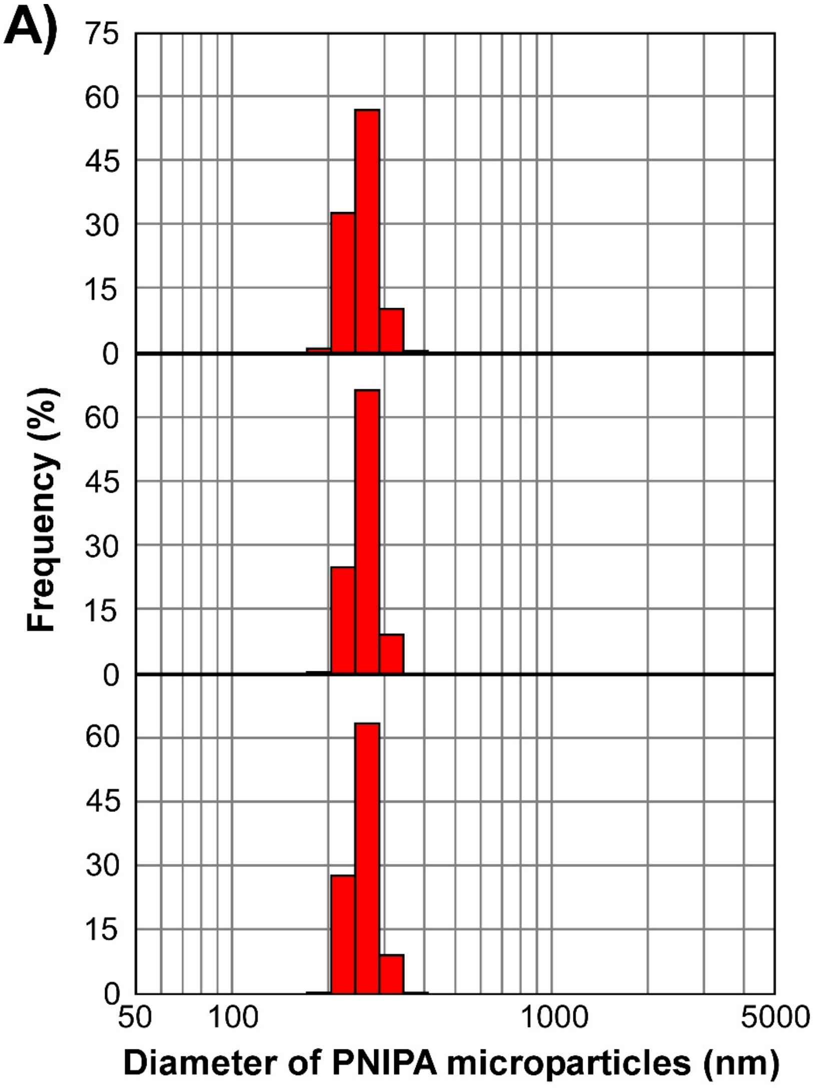

B)

Times of reuse experiment	Mean diameter of PNIPA microparticles at 20 °C (nm)	CV in diameter of PNIPA microparticles at 20 °C (%)
Original	256	11.0
1st time	258	9.8
2nd time	259	9.3

Figure S6. Size properties of **PNIPA-5**, analyzed by the DLS measurements at 20 ºC, before and after reuse experiments. A) Size distribution profiles of **PNIPA-5** before (upper panel) and after the first (middle panel) and second (lower panel) reuse experiments. B) List of mean diameters and their CVs of **PNIPA-5** before and after the reuse experiments.

3. Supporting Movies

Movie S1: Color switching of dialyzed suspensions of PNIPA hydrogel microparticles between reflection color of red or green and white turbidity upon heating and cooling processes (MP4).

Movie S2: Comparison of visual appearance for two kinds of suspensions of PNIPA hydrogel microparticles, purified by dialysis or combination of centrifugation and deionization, containing small amounts of carbon black nanoparticles upon heating and cooling processes (MP4).
